# Targeting YBX1‐m5C mediates *RNF115* mRNA circularisation and translation to enhance vulnerability of ferroptosis in hepatocellular carcinoma

**DOI:** 10.1002/ctm2.70270

**Published:** 2025-03-15

**Authors:** Ouwen Li, Ke An, Han Wang, Xianbin Li, Yueqin Wang, Lan Huang, Yue Du, Nuo Qin, Jiasheng Dong, Jingyao Wei, Ranran Sun, Yong Shi, Yanjia Guo, Xiangyi Sun, Ying Yang, Yun‐Gui Yang, Quancheng Kan, Xin Tian

**Affiliations:** ^1^ Department of Pharmacy The First Affiliated Hospital of Zhengzhou University Zhengzhou Henan China; ^2^ Henan Key Laboratory of Precision Clinical Pharmacy Zhengzhou University Zhengzhou China; ^3^ Translational Medicine Center The First Affiliated Hospital of Zhengzhou University Zhengzhou Henan China; ^4^ Department of Infectious Diseases The First Affiliated Hospital of Zhengzhou University Zhengzhou Henan China; ^5^ Gene Hospital of Henan Province Precision Medicine Center The First Affiliated Hospital of Zhengzhou University Zhengzhou Henan China; ^6^ Key Laboratory of Genomic and Precision Medicine, Collaborative Innovation Center of Genetics and Development, Beijing Institute of Genomics, Chinese Academy of Sciences China National Center for Bioinformation Beijing China

**Keywords:** 5‐methylcytosine modification, ferroptosis, hepatocellular carcinoma, translational regulation, YBX1

## Abstract

**Background:**

RNA 5‐methylcytosine (m5C) plays an important role in the progression of hepatocellular carcinoma (HCC). Dysregulation of ferroptosis is closely associated with HCC. However, the effect of the epigenetic mRNA m5C modification on ferroptosis in HCC remains unclear.

**Methods:**

In this study, ferroptosis was evaluated by detecting lipid reactive oxygen species (lipid ROS), ferrous ion and 4‐hydroxynonenal (4‐HNE) in xenograft mouse model, diethylnitrosamine (DEN)‐initiated HCC model and so forth. The regulatory mechanisms of YBX1 in mRNA translation were elucidated using RNA sequencing, ribosome sequencing, RNA immunoprecipitation (RIP)‐sequencing, bisulphite sequencing and immunoprecipitation (IP)–mass spectrometry assays. Dual‐luciferase reporter, RIP‐qPCR, Co‐IP, RNA pulldown and methylated RNA immunoprecipitation (MeRIP)‐quantitative polymerase chain reaction (qPCR) assays were performed to validate the mechanism of YBX1 in regulating mRNA translation by m5C modification.

**Results:**

Here, we found that YBX1 promoted the translation of Ring Finger Protein 115 (RNF115) mRNA through m5C modification, thereby inhibiting ferroptosis and promoting HCC development. Moreover, RNF115 was identified as an E3 ubiquitin ligase for dihydroorotate dehydrogenase (DHODH), promoting Lys27 (K27) ubiquitination and inhibiting its autophagic degradation to counteract ferroptosis. In addition, YBX1 bound to the m5C modification sites of *RNF115* 3′‐untranslated region (UTR) and interacted with Eukaryotic Translation Initiation Factor 4A1 (EIF4A1) to bridge the 5′‐UTR regions, promoting mRNA circularisation and translation, while NOP2/Sun RNA methyltransferase 2 (NSUN2) was identified as responsible for m5C modification of *RNF115* mRNA in HCC.

**Conclusions:**

The current work revealed that YBX1 promoted *RNF115* mRNA translation in an m5C‐dependent manner, thereby regulating DHODH ubiquitination and expression to suppress ferroptosis. This research sheds light on the mechanism of YBX1 in m5C‐modified mRNAs translation and ferroptosis, highlighting its promise as a biomarker for prognosis and a target for therapy in HCC.

**Key points:**

- YBX1 inhibits ferroptosis in HCC by regulating the RNF115‐DHODH axis.
- RNF115, an E3 ligase, mediates K27 ubiquitination and autophagic degradation of DHODH.
- YBX1 binds to the m5C sites of RNF115 mRNA 3′‐UTR and interacts with EIF4A1 to bridge the 5′‐UTR, promoting mRNA circularisation and translation.
- High expression of YBX1/RNF115 predicts the poor overall survival in HCC.

## Graphical abstract

NOP2/Sun RNA methyltransferase 2 (NSUN2) promotes 5‐methylcytosine (m5C) modification of Ring Finger Protein 115 (RNF115) mRNA, which is subsequently bound by YBX1. Then, YBX1 interacts with Eukaryotic Translation Initiation Factor 4A1 (EIF4A1) to promote *RNF115* mRNA looping and translation. Subsequently, RNF115 interacts with dihydroorotate dehydrogenase (DHODH) to promote its K27 ubiquitination and inhibit the autophagic degradation of DHODH to resist ferroptosis and promote hepatocellular carcinoma (HCC) progression.

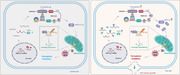

## 1 INTRODUCTION

Liver cancer is among the most prevalent malignancies globally.^1^, ^2^, ^3^ Hepatocellular carcinoma (HCC) represents approximately 90% of all cases of primary liver cancer,^4^ and the overall 5‐year survival rate is about 18%.^5^ RNA methylation modifications play a key role in the occurrence and progression of various cancers.^6^ 5‐Methylcytosine (m5C) modification is a prevalent form of RNA modification, widely present in various RNA species.^7^ In addition to regulating mRNA stability, m5C modification modulates the nuclear export and translation of mRNAs.^8^, ^9^ Dysregulated m5C methylation of mRNA promotes tumour progression by altering its epigenetic features.^9^, ^10^, ^11^ Our previous study identified that the primary m5C methyltransferase NOP2/Sun RNA methyltransferase 2 (NSUN2) promoted *GRB2* mRNA m5C methylation, thereby contributing to the progression of HCC.^12^ The main m5C reader proteins include ALYREF and YBX1, but their biological functions differ. ALYREF primarily mediates the nuclear export of mRNA, and its role in HCC progression has been reported.^13^ In contrast to ALYREF, YBX1, which functions as a ‘reader’ of m5C modification, recognises m5C‐modified mRNA through the indole ring of W65 in its cold shock domain, enhancing mRNA stability.^14^ However, the YBX1‐W65A mutant cannot bind to m5C‐modified mRNA.^15^ The upregulated expression of YBX1 in various tumours is associated with poor prognosis.^14^ However, additional research is required to explore the biological functions of YBX1 beyond its role in regulating mRNA stability and promoting tumour progression. Remarkably, the level of m5C modification was positively associated with mRNA translation.^8^ Recent studies have demonstrated that YBX1 regulates the translation of specific mRNAs. However, the role of m5C modification in the YBX1‐mediated translational regulation is unclear.^16^ Therefore, elucidating the biological function of YBX1 in tumour progression could provide a promising foundation for therapeutic strategies.

Current studies have reported that ferroptosis is involved in HCC development and therapeutic response.^17^, ^18^, ^19^, ^20^ Ferroptosis is a lipid peroxidation process dependent on iron ions.^21^, ^22^, ^23^, ^24^ Several small‐molecule inducers of ferroptosis that target the clearance of lipid peroxides have been identified. For example, erastin regulates cystine/glutathione (GSH) transport, RSL3 directly inhibits glutathione peroxidase 4 (GPX4), iFSP1 and FIN56 inhibit Coenzyme Q10 (CoQ)‐CoQH_2_ transformation.^25^ However, the critical signalling pathways and epigenetic regulators that mediate ferroptosis remain elusive. Several proteins from the NSUN protein family are associated with ferroptosis.^26^ However, further studies are needed to determine whether m5C modification mediates tumour progression through the regulation of ferroptosis.

In this study, YBX1 promoted HCC progression by inhibiting ferroptosis through the upregulation of Ring Finger Protein 115 (RNF115) mRNA translation. Moreover, we found that RNF115 as a novel E3 ubiquitin ligase of dihydroorotate dehydrogenase (DHODH) that mediated K27 ubiquitination and autophagic degradation of DHODH, thereby protecting against ferroptosis. Furthermore, YBX1 could bind to the m5C modification sites of *RNF115* 3′‐untranslated region (UTR) and interacted with Eukaryotic Translation Initiation Factor 4A1 (EIF4A1) to bridge the 5′‐UTR, promoting mRNA circularisation and translation. Additionally, NSUN2 was identified as a methyltransferase that catalysed m5C modifications in *RNF115* mRNA. Therefore, this work provided a potential therapeutic strategy to inhibit HCC progression by abrogating YBX1 to promote ferroptosis response.

## 2 RESULTS

### 2.1 YBX1 is highly expressed in HCC and is associated with poor prognosis

**FIGURE 1:**
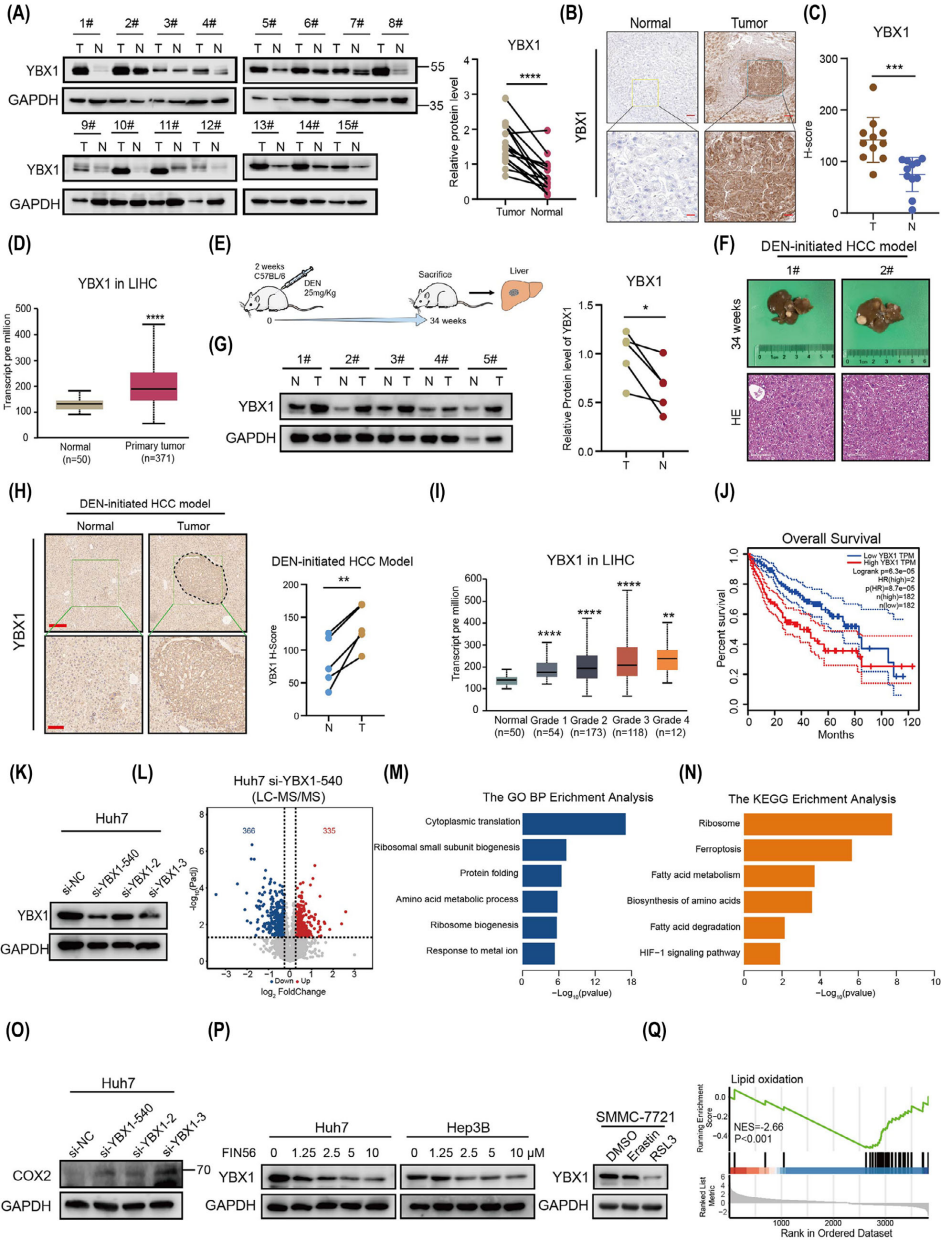
YBX1 is highly expressed in hepatocellular carcinoma (HCC) and is associated with poor prognosis. (A) Western blot was used to analyse the expression of YBX1 in 15 pairs of HCC tissue samples and statistical analysis (****p < .0001, paired *t*‐test. T, tumour. N, normal). (B, C) YBX1 immunohistochemical (IHC) staining and statistical analysis (T, tumour = 11, N, normal = 11, unpaired *t*‐test). Scale bar: 100 µm (upper panel) and 50 µm (lower panel). (D) YBX1 expression levels were analysed by The Cancer Genome Atlas (TCGA) database. (E–H) Construction of diethylnitrosamine (DEN)‐induced HCC model (E), haematoxylin–eosin (H&E) staining image (scale bar, 100 µm) (F), western blotting (WB) and IHC detection of YBX1 expression (G, H; *p < .05, **p < .01, paired *t*‐test, scale bar: 100 µm (upper panel) and 50 µm (lower panel)). (I) YBX1 expression levels in different grades of progress by TCGA database analysis. (J) Correlation analysis of YBX1 expression levels with overall survival using GEPIA database (http://gepia.cancer‐pku.cn/). (K, L) The efficiency of YBX1 knockdown was verified in Huh7 by WB (K) and liquid chromatography with tandem mass spectrometry (LC–MS/MS) analysis of differential protein volcano map (L). (M, N) Gene Ontology (GO) and Kyoto Encyclopedia of Genes and Genomes (KEGG) pathway enrichment analysis of downregulated proteins after knockdown YBX1 in Huh7. (O) COX2 protein level in the Huh7 knockdown cells. (P) Protein expression of YBX1 after treatment with FIN56, erastin (10 µM) or RSL3 (10 µM) for 24 h in HCC cells. (Q) The relationship between YBX1 and lipid oxidation pathways was analysed by GSEA in TCGA database.

Our previous research has shown that dysregulated mRNA m5C modification promotes HCC progression.^12^ However, the role of the m5C reader YBX1 in dysregulated mRNA m5C modification‐mediated HCC progression is unclear. To investigate the role of YBX1 in HCC development, HCC tissues were subjected to western blotting (WB) and immunohistochemical (IHC) analyses. Additionally, the HCC datasets from The Cancer Genome Atlas (TCGA) database were analysed, revealing that YBX1 expression was upregulated in HCC (Figure 1A–D). Next, a diethylnitrosamine (DEN)‐initiated HCC model was constructed using C57BL/6 mice. YBX1 expression in tumour tissues was markedly increased compared to normal tissues (Figure 1E–H). TCGA data analysis revealed that YBX1 expression was upregulated with the progression of HCC, and high YBX1 expression was strongly correlated with poor prognosis (Figure 1I, J). To elucidate the pivotal role of YBX1 in the initiation and progression of HCC, we used three different small interfering RNAs (si‐RNAs) targeting YBX1 and performed liquid chromatography with tandem mass spectrometry (LC–MS/MS) analysis (Figures 1K, L and S1A). Gene Ontology (GO) pathway enrichment analysis revealed that the downregulated proteins were significantly associated with cytoplasmic translation and ribosome biogenesis signalling. Meanwhile, Kyoto Encyclopedia of Genes and Genomes (KEGG) pathway analysis indicated that ferroptosis may be a key signalling pathway mediated by YBX1 (Figures 1M, N and S1B, C). *PTGS2*, a ferroptosis biomarker that encodes the COX2 protein,^27^, ^28^, ^29^, ^30^ was significantly downregulated in the tumour tissues of the DEN‐initiated HCC mouse model. This suggested that the ferroptosis signalling pathway is significantly suppressed in HCC (Figure S1D). However, knockdown of YBX1 upregulated COX2 expression (Figure 1O), suggesting that knockdown of YBX1 may reverse the suppressed state of ferroptosis. To examine the biological role of YBX1 in ferroptosis, HCC cell lines were treated with three ferroptosis activators (erastin, RSL3 and FIN56). Consistent with previous findings, treatment with erastin, RSL3 and FIN56 increased lipid ROS and inhibited cell proliferation in Hep3B, Huh7 and SMMC‐7721 cells. However, treatment with ferrostatin‐1 (Fer‐1), a ferroptosis inhibitor, mitigated the ferroptosis activator‐induced changes in lipid ROS and cell proliferation (Figure S1E–G). It was noteworthy that ferroptosis inducers significantly downregulated the protein expression of YBX1, suggesting that YBX1 may be an inhibitor of ferroptosis (Figure 1P). Analysis of TCGA datasets revealed that YBX1 expression was negatively correlated with the lipid oxidation signalling pathway (Figure 1Q). These results indicate that YBX1 is significantly overexpressed in HCC and may promote the progression of HCC by inhibiting ferroptosis.

### 2.2 YBX1 suppresses ferroptosis of HCC in vitro and in vivo

**FIGURE 2:**
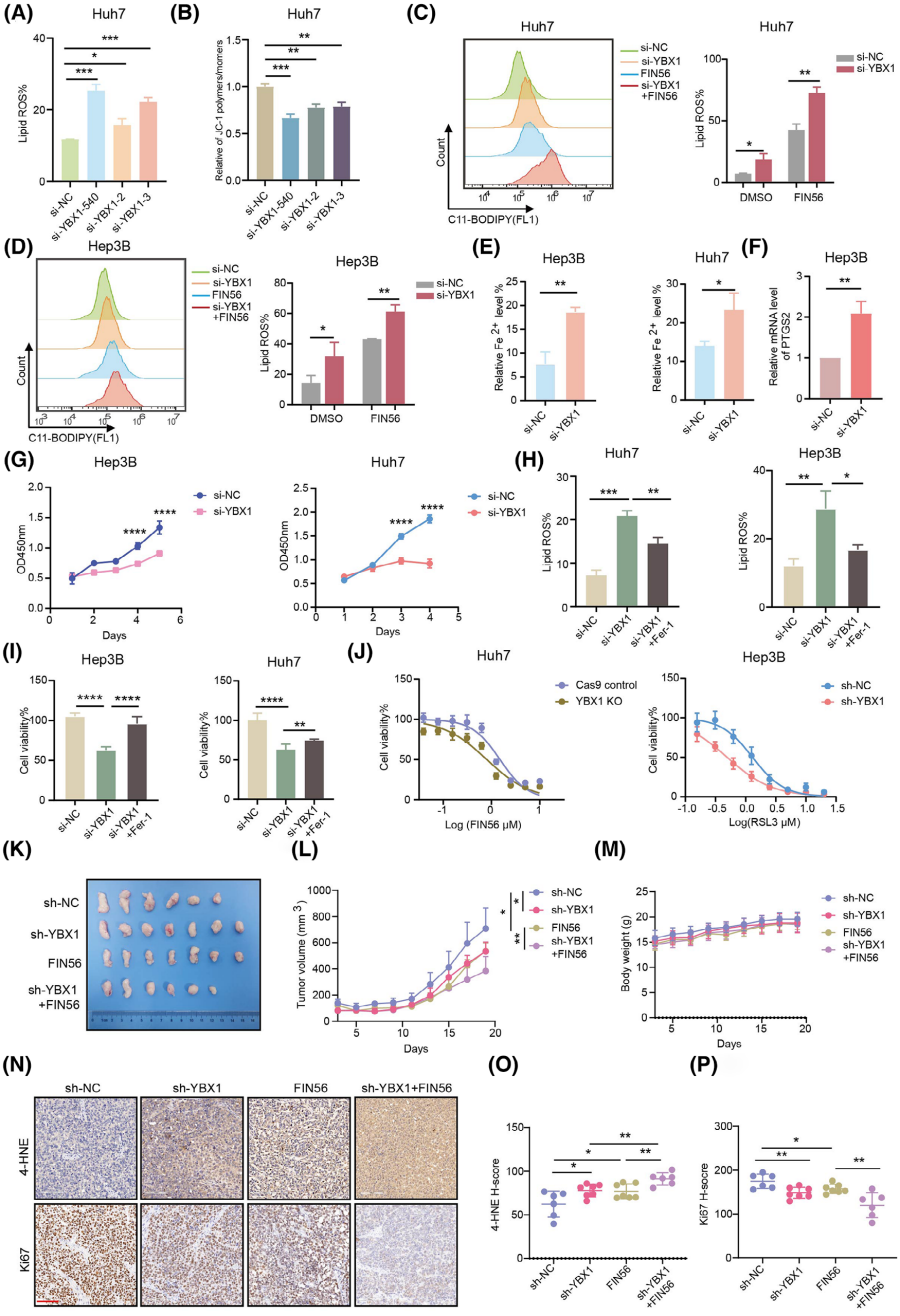
YBX1 negatively regulates ferroptosis of hepatocellular carcinoma (HCC) in vitro and in vivo. (A, B) Lipid reactive oxygen species (ROS) levels (A) and mitochondrial membrane potential (B) were assessed by flow cytometry following YBX1 knockdown. (C, D) Huh7 and Hep3B cells transfected with si‐YBX1 or si‐NC for 48 h were exposed to FIN56 (5 µM) for 8 h and lipid ROS was detected by flow cytometry. (E–G) After YBX1 knockdown, Fe^2+^ loading (E), *PTGS2* mRNA (F) and cell proliferation (G) were detected. (H, I) YBX1 knockdown combined with Fer‐1 treatment was used to detect lipid ROS level (Fer‐1: 1 µM, 8 h) and cell viability (Fer‐1: 50 nM in Hep3B or 200 nM in Huh7 for 72 h). (J) Huh7 knockout YBX1 or Hep3B knockdown YBX1 cells were treated with FIN56 (24 h) or RSL3 (72 h) at varying concentrations, and IC50 was determined using a Cell Counting Kit‐8 (CCK8) assay. (K–M) Tumour image (K), tumour volume (L) and mice body weight (M) of xenografts subcutaneously implanted with SMMC‐7721 sh‐NC, sh‐YBX1 cells in BALB/c nude mice (*n* = 6 or 7 per group). (N–P) Representative images (N) and statistical analysis of 4‐hydroxynonenal (4‐HNE) (O), Ki67 (P) immunohistochemical (IHC) staining obtained from tumour xenografts. Scale bar, 100 µm. Data are shown as mean ± standard deviation (SD), *p < .05, **p < .01, ***p < .001, ****p < .0001. Unpaired *t*‐test was used unless otherwise stated.

To demonstrate the regulatory role of YBX1 in ferroptosis in HCC, we used three different si‐RNAs targeting YBX1 and found that they significantly increased the production of lipid reactive oxygen species (ROS) and facilitated the dissipation of mitochondrial membrane potential (Figures 2A, B and S2A–C). The si‐YBX1‐540 construct (referred to as si‐YBX1 hereafter) was used in all subsequent experiments as it exhibited the highest knockdown efficiency (Figure 1K). Knockdown of YBX1 increased lipid ROS accumulation, which was further enhanced by treatment with FIN56, erastin or RSL3 (Figures 2C, D and S2D–F). Moreover, YBX1 knockdown promoted Fe^2+^ loading, increased *PTGS2* mRNA and COX2 protein expression, and inhibited cell proliferation (Figures 2E–G and S2G–J). Treatment with the ferroptosis inhibitor Fer‐1 mitigated the YBX1 knockdown‐induced lipid ROS accumulation and cell death (Figures 2H, I and S2K). Additionally, YBX1 knockdown significantly enhanced the sensitivity of Huh7 and Hep3B cells to FIN56 and RSL3 (Figures 2J and S2L, M). However, overexpression of YBX1 significantly inhibited the levels of lipid ROS, Fe^2+^ loading, *PTGS2* mRNA expression, and promoted the proliferation of Hpe3B, Huh7 and SMMC‐7721 cells and resisted the sensitivity to FIN56 and RSL3 (Figure S3A–F). These results demonstrated that YBX1 negatively regulated ferroptosis in vitro in HCC cells.

In vivo, YBX1 knockdown and FIN56 treatment synergistically decreased the volume of xenografts (Figure 2K–M). However, overexpression of YBX1 significantly promoted the proliferation of tumour xenografts (Figure S3G). IHC analysis showed that YBX1 knockdown increased the accumulation of ferroptosis marker 4‐hydroxynonenal (4‐HNE) and inhibited the expression of Ki67. Overexpression of YBX1 resulted in the opposite effect (Figures 2N–P and S3H, I).

Together, these results demonstrate that YBX1 inhibits ferroptosis of HCC in vivo and in vitro.

### 2.3 RNF115 is the target of YBX1 in regulating ferroptosis

**FIGURE 3:**
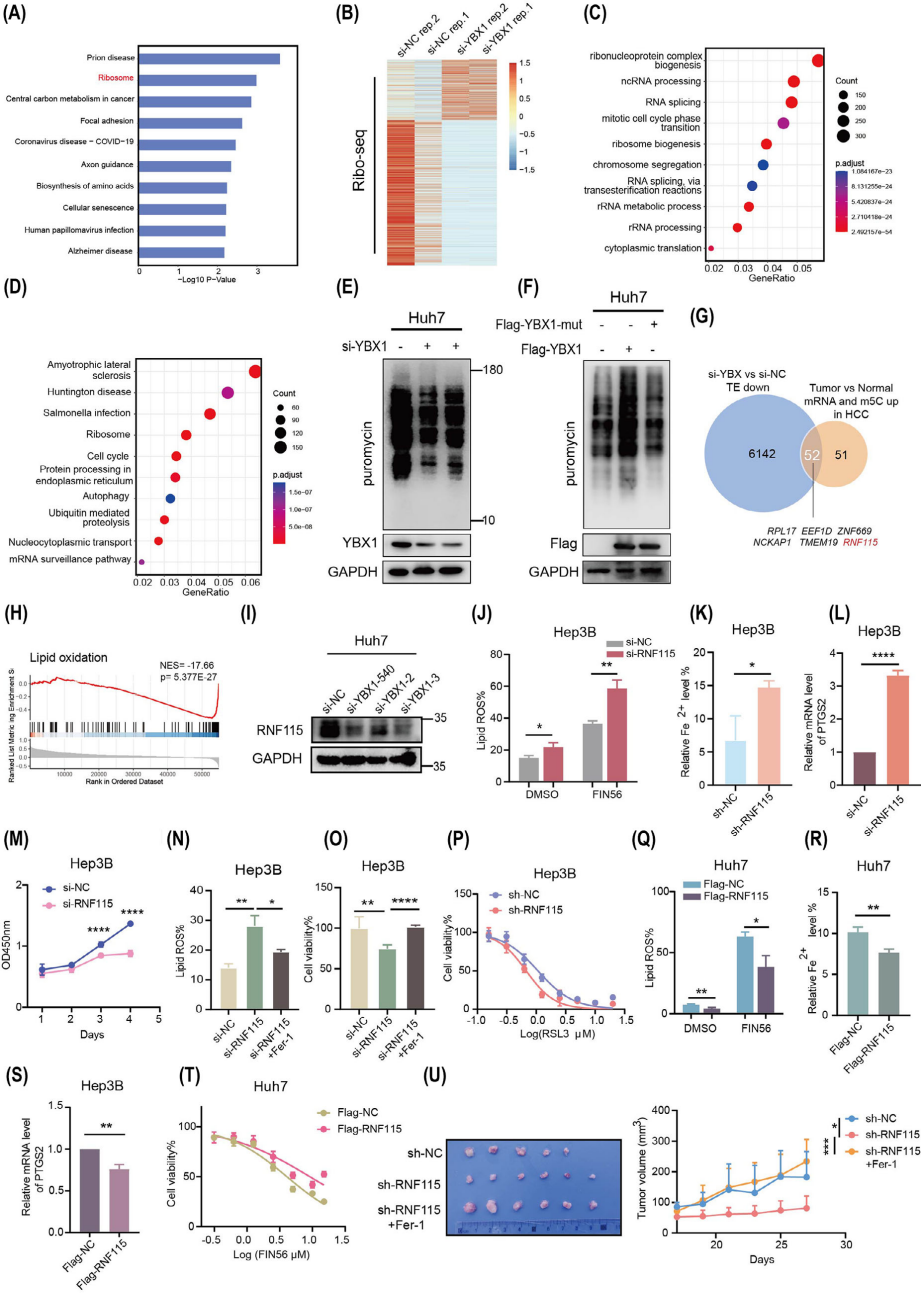
Ring Finger Protein 115 (RNF115) is the target of YBX1 in regulating ferroptosis. (A) Kyoto Encyclopedia of Genes and Genomes (KEGG) pathway analysis was used to analyse the downregulated genes in YBX1 knockdown by RNA‐seq. (B) Heatmap showed the genes with altered translation efficiency (TE) after YBX1 knockdown in SMMC‐7721 by Ribo‐seq. (C, D) Gene Ontology (GO) and KEGG showed the genes with altered TE after YBX1 knockdown by Ribo‐seq. (E, F) In Huh7 cells, YBX1 knockdown or overexpression of YBX1‐wt and YBX1‐mut (W65A) was assessed for protein synthesis by western blotting (WB). (G) The Venn diagram illustrated the intersection of genes showing mRNA upregulation and 5‐methylcytosine (m5C) methylation upregulation in hepatocellular carcinoma (HCC) tumour tissue with those exhibiting decreased TE in YBX1 knockdown SMMC‐7721 cells. (H) Correlation analysis of RNF115 with lipid oxidation signalling by GSEA in The Cancer Genome Atlas (TCGA) database. (I) RNF115 expression was detected by WB after 48 h of YBX1 knockdown by si‐RNA. (J) Hep3B cells transfected with si‐RNF115 or si‐NC for 48 h were exposed to FIN56 for 8 h and lipid reactive oxygen species (ROS) was detected by flow cytometry. (K–M) Fe^2+^ loading, *PTGS2* mRNA levels and cell proliferation were detected after RNF115 knockdown. (N, O) After RNF115 knockdown in Hep3B cells, combined treatment with Fer‐1 was used to assess lipid ROS levels (Fer‐1: 1 µM for 8 h) and cell viability (Fer‐1: 15 nM for 72 h). (P) The IC50 of RSL3 (72 h) was measured by CCK8 assay in Hep3B cells stably transfected with sh‐RNF115. (Q) Huh7 cells stably transfected with Flag‐RNF115 or Flag‐NC were exposed to FIN56 (5 µM) for 8 h and lipid ROS was detected by flow cytometry. (R–T) Fe^2+^ loading, *PTGS2* mRNA levels and IC50 of FIN56 (24 h) were detected in stable expression of Flag‐RNF115 or Flag‐NC cells. (U) Tumour image and tumour volume of xenografts subcutaneously implanted with Huh7 sh‐NC and sh‐RNF115 cells in BALB/c nude mice (*n* = 5 or 6 per group). Data are shown as mean ± standard deviation (SD), *p < .05, **p < .01, ***p < .001, ****p < .0001. Unpaired *t*‐test was used unless otherwise stated.

To investigate the mechanism underlying YBX1‐mediated ferroptosis in HCC, the KEGG analysis of the RNA‐seq data showed that YBX1 knockdown was associated with the ribosome signalling pathway (Figure 3A). GO pathway enrichment analysis of LC–MS/MS data also revealed a significant association with translational signalling pathways (Figures 1M and S1B). It has been reported that targeting translation is considered an important strategy for tumour treatment.^31^ To further investigate the regulation of YBX1 on translation, ribosome sequencing (Ribo‐seq) analyses were conducted in SMMC‐7721 cells. The translation efficiency of 6194 genes was downregulated in YBX1 knockdown cells (Figure 3B). GO and KEGG analysis demonstrated that these genes with downregulated translation efficiency were enriched in the regulation of translation (ribonucleoprotein complex biogenesis, ribosome biogenesis and rRNA process signalling pathways; Figure 3C, D). Puromycin intake assay also elucidated that YBX1 knockdown inhibited global protein synthesis (Figures 3E and S4A). Notably, overexpression of YBX1‐wt, but not the YBX1‐W65A mutant (with mutant YBX1 m5C binding site), significantly promoted global protein synthesis (Figure 3F). This suggested that YBX1 regulation of global protein synthesis depended on m5C modification. Next, to explore how YBX1 regulates key target genes involved in translation‐mediated ferroptosis through m5C modification. We tried to identify downstream genes that were significantly upregulated at both the mRNA and m5C modification levels in HCC tumour tissues, and strongly regulated by YBX1 at the translational level. We found that *RNF115* and *ZNF669* were among the top 10 genes in the intersection set and were significantly negatively correlated with the lipid oxidation signal (Figures 3G, H and S4B). However, knockdown of YBX1 significantly downregulated RNF115 expression but not ZNF669 (Figures 3I and S4C, D). Two specific si‐RNA constructs targeting RNF115 were used for its knockdown, both of which significantly promoted lipid ROS accumulation and reduced mitochondrial membrane potential (Figure S4E–G). As both si‐RNA constructs exhibited similar RNF115 knockdown efficiency, subsequent experiments were performed using the si‐RNF115‐1 construct (referred to as si‐RNF115 hereafter). Treatment with FIN56, erastin and RSL3 promoted the RNF115 knockdown‐induced accumulation of lipid ROS (Figures 3J and S4H, I). Furthermore, RNF115 knockdown promoted Fe^2+^ loading, increased *PTGS2* mRNA and COX2 protein expression and inhibited cell proliferation, while Fer‐1 reversed lipid ROS accumulation and cell death by RNF115 knockdown (Figures 3K–O and S4J–P). Additionally, RNF115 knockdown enhanced the sensitivity of Huh7 and Hep3B cells to FIN56 and RSL3 (Figures 3P and S4Q). However, overexpression of RNF115 demonstrated opposite effects on ferroptosis (Figures 3Q–T and S5A–E). These results illustrated that RNF115 repressed ferroptosis in HCC in vitro. In vivo, overexpression of RNF115 significantly promoted the proliferative capacity of xenograft tumours (Figure S5F). In contrast, knockdown of RNF115 inhibited tumour proliferation, and this effect was rescued by Fer‐1, suggesting that RNF115 inhibits ferroptosis in vivo (Figure 3U).

To elucidate the role of RNF115 in YBX1‐mediated ferroptosis, we found that RNF115 knockdown reversed the downregulation of lipid ROS caused by overexpression of YBX1, with or without FIN56 treatment in Huh7 cells (Figure S5G). Moreover, RNF115 overexpression inhibited the accumulation of lipid ROS induced by YBX1 knockdown in SMMC‐7721, Huh7 and Hep3B cells (Figure S5H, I). Our findings indicate that RNF115 is a downstream target of YBX1 for inhibiting ferroptosis in HCC.

### 2.4 RNF115 suppresses ferroptosis by promoting the K27 ubiquitination and autophagic degradation of DHODH

**FIGURE 4:**
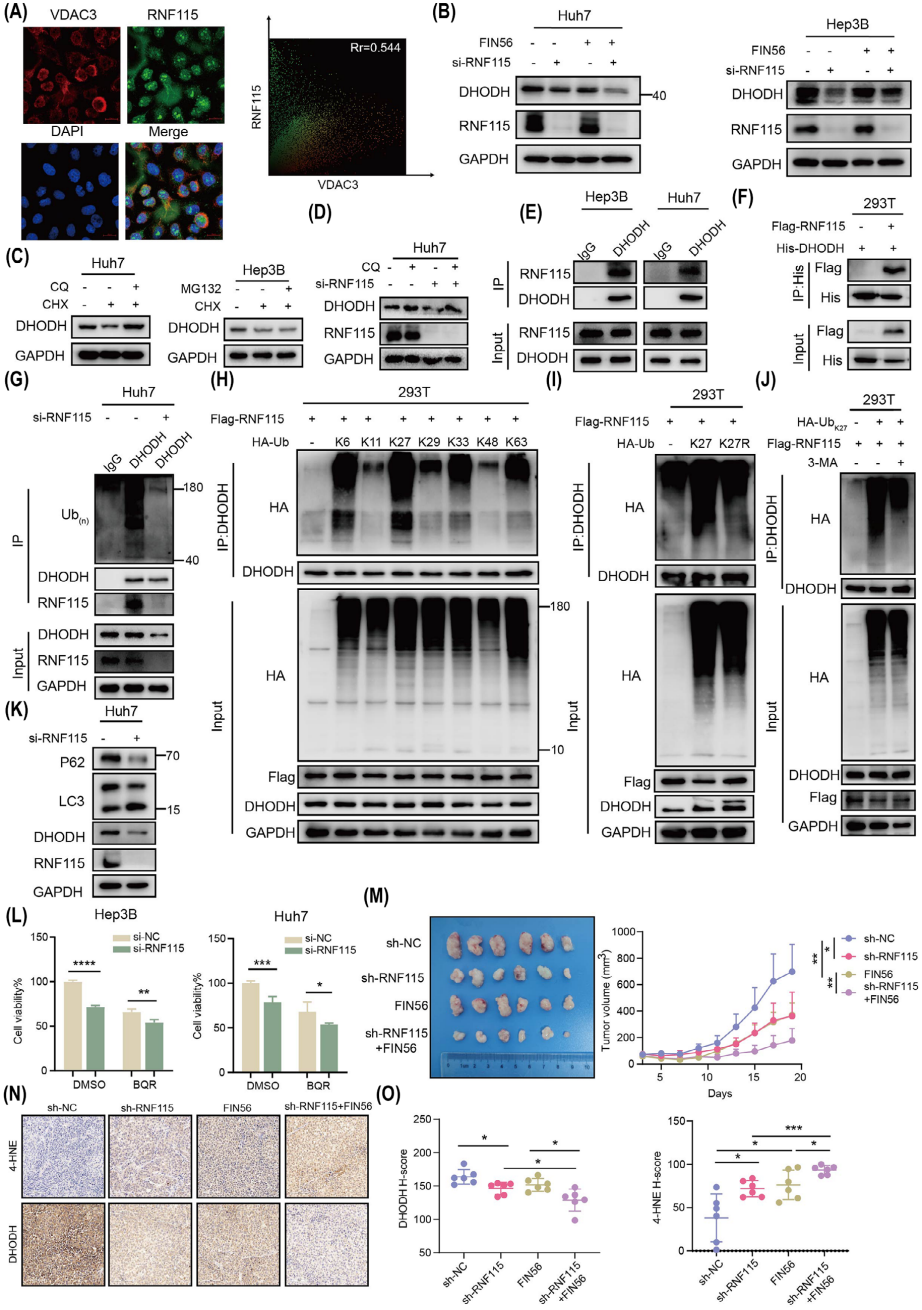
Ring Finger Protein 115 (RNF115) suppresses ferroptosis by promoting the K27 ubiquitination and autophagic degradation of dihydroorotate dehydrogenase (DHODH). (A) Co‐localisation of Voltage Dependent Anion Channel 3 (VDAC3) with RNF115 was analysed by immunofluorescence (IF) in SMMC‐7721 cells. (B) After knockdown of RNF115 in Huh7 and Hep3B cells for 48 h by si‐RNA, the expression of DHODH was detected by western blotting (WB) with or without FIN56 (2.5 µM, 24 h) treatment. (C) After combined treatment with chlorhexidine (CHX; 40 µM) and MG132 (20 µM) or chloroquine (CQ; 20 µM) for 24 h, the protein expression of DHODH was detected by WB. (D) After knockdown RNF115 in Huh7 cells and combined treatment with CQ (20 µM), the protein expression of DHODH was detected by WB. (E, F) Co‐immunoprecipitation (Co‐IP) was used to detect endogenous (E) or exogenous (F) DHODH interacting with RNF115. (G) After knockdown RNF115 in Huh7 cells, the ubiquitination level of DHODH was detected by Co‐IP. (H, I) HEK293T cells were transfected with Flag‐RNF115 and HA‐Ub (Lys6, Lys11, Lys27, Lys29, Lys33, Lys48 or Lys63 only) as indicated (H); HEK293T cells were transfected with Flag‐RNF115 and HA‐Ub (Lys27; K27R) as indicated (I). (J) DHODH ubiquitination modification was detected in HEK293T cells transfected with Flag‐RNF115, HA‐Ub_K27_ in combination with 3‐methyladenine (3‐MA; 5 mM, 24 h) treatment. (K) WB was used to detect the expression levels of autophagy‐related proteins after knockdown RNF115. (L) Hep3B and Huh7 cells transfected with si‐RNF115 and treated with Brequinar (BQR, 5 µM) for 48 h, and cell viability was detected by CCK8. (M) Tumour image and tumour volume of xenografts subcutaneously implanted with SMMC‐7721 sh‐NC and sh‐RNF115 cells in BALB/c nude mice (*n* = 6 per group). (N, O) Representative images (N) and statistical analysis (O) of 4‐hydroxynonenal (4‐HNE) and DHODH immunohistochemical (IHC) staining obtained from tumour xenografts. Scale bar, 100 µm. Data are shown as mean ± standard deviation (SD), *p < .05, **p < .01, ***p < .001, ****p < .0001. Unpaired *t*‐test was used unless otherwise stated.

Next, to further explore the mechanism of RNF115 inhibiting HCC ferroptosis, *The Human Protein Atlas* database suggested that RNF115 was mainly localised in nucleolus and mitochondria (Figure S6A). Consistently, immunofluorescence (IF) analysis also showed that RNF115 co‐localised with mitochondria outer membrane protein Voltage Dependent Anion Channel 3 (VDAC3; Figure 4A). It is worth noting that mitochondrial fate is inextricably linked to ferroptosis.^32^ This suggested that RNF115 may mediate ferroptosis inhibition in mitochondria. Notably, the known mitochondrial proteins involved in resisting ferroptosis include GPX4 and DHODH.^33^ Moreover, both knockdown of YBX1 and RNF115 downregulated the expression of DHODH, but not GPX4, with or without FIN56 treatment (Figures 4B and S6B, C). To investigate whether YBX1/RNF115 affect other ferroptosis‐related signalling pathways, we knocked down YBX1/RNF115 and assessed the expression levels of SLC7A11 (a cystine/glutamate transporter), FSP1 (a cytoplasmic CoQ–CoQH_2_ axis‐related protein) and ACSL3 (a fatty acid metabolism signalling‐related protein). Knockdown of YBX1/RNF115 slightly affected the expression of SLC7A11, FSP1 and ACSL3, but significantly downregulated the expression of DHODH (Figure S6D, E). Interestingly, neither knockdown nor overexpression of RNF115 affected the mRNA levels of *DHODH*, suggesting that RNF115 may mediate the expression of DHODH through post‐translational modification (Figure S6F, G). Notably, RNF115 functions as an E3 ubiquitin ligase. The autophagy inhibitors chloroquine (CQ) and 3‐methyladenine (3‐MA), but not the proteasome inhibitor MG132, enhanced the stability of DHODH in cells treated with chlorhexidine (CHX), a protein synthesis inhibitor (Figures 4C and S6H). Additionally, CQ and 3‐MA reversed the RNF115 knockdown‐induced downregulation of DHODH, suggesting that RNF115 regulates the autophagic degradation of DHODH (Figures 4D and S6I). Furthermore, endogenous and exogenous co‐immunoprecipitation (Co‐IP) experiments revealed that RNF115 strongly interacted with DHODH (Figure 4E, F). To further investigate whether RNF115 mediates the ubiquitination of DHODH, we observed a reduced ubiquitination level of DHODH upon RNF115 knockdown in Huh7 and Hep3B cells (Figures 4G and S6J). Further investigation revealed that RNF115 primarily mediated the Lys6 (K6) and Lys27 (K27) ubiquitination of DHODH (Figure 4H). The K27 ubiquitination modification is closely associated with autophagy,^34^, ^35^ and RNF115 promoted the K27 ubiquitin chain of DHODH, but not the Lys27‐to‐Arg (K27R) mutant (Figure 4I). Moreover, we found that only the wild‐type RNF115 (RNF115‐wt), not the catalytically inactive mutant RNF115 (RNF115‐2CA, C228A/C231A), promoted the K27 ubiquitin chain modification of DHODH (Figure S6K). This suggested that the RNF115‐mediated K27 ubiquitination of DHODH is dependent on RNF115's ubiquitin ligase activity. To further investigate the relationship between the K27 ubiquitination of DHODH and autophagy, we found that 3‐MA significantly inhibited the K27 ubiquitin chain modification of DHODH (Figure 4J). This suggested that the K27 ubiquitination modification of DHODH suppressed its autophagic degradation. Moreover, knockdown of RNF115 significantly increased LC3 II expression and downregulated P62 expression, thereby promoting autophagy in Huh7 and Hep3B cells (Figures 4K and S6L). In summary, these results demonstrate that RNF115 acts as an E3 ubiquitin ligase for DHODH, mediating its K27 ubiquitin modification and autophagic degradation.

To further investigate if YBX1/RNF115‐mediated ferroptosis in HCC via DHODH, two independent si‐RNA constructs were used to specifically target DHODH. Knockdown of DHODH significantly promoted the accumulation of lipid ROS, decreased the mitochondrial membrane potential and inhibited cell proliferation (Figure S7A–D). As both si‐RNA constructs exhibited similar DHODH knockdown efficiency, we used si‐DHODH‐1 in all subsequent experiments (referred to as si‐DHODH hereafter). Brequinar (BQR), an inhibitor of DHODH, synergistically promoted the accumulation of lipid ROS, suppressed mitochondrial membrane potential and inhibited cell viability when combined with the knockdown of YBX1 or RNF115 (Figures 4L and S7E–H). However, exogenous supplementation of Uridine did not reverse this effect, indicating independence from the de novo pyrimidine synthesis pathway (Figure S7I). In contrast, si‐DHODH transfection or BQR treatment partially suppressed the YBX1 or RNF115 overexpression‐induced downregulation of lipid ROS and upregulation of mitochondrial membrane potential and cell proliferation (Figure S7J–R). However, DHODH reintroduction significantly alleviated the accumulation of lipid ROS caused by YBX1/RNF115 knockdown (Figure S7S, T).

In vivo, YBX1/RNF115 knockdown combined with FIN56 synergistically reduced xenograft volume (Figures 2L and 4M). Importantly, knockdown of YBX1/RNF115 together with FIN56 treatment promoted the accumulation of 4‐HNE and downregulated DHODH expression (Figures 2N, O, 4N, O and S8A, B). However, in xenografts with YBX1 overexpression, the expression of RNF115 and DHODH was significantly upregulated (Figure S8C, D). Additionally, reconstitution of RNF115 during YBX1 knockdown significantly reversed the inhibitory effect of YBX1 knockdown on xenograft volume and weight and notably reversed the expression level of Ki67, 4‐HNE and DHODH (Figure S8E–H). In summary, these results suggest that RNF115 inhibits ferroptosis by functioning as an E3 ubiquitin ligase of DHODH and promoting its K27 ubiquitination and autophagic degradation.

### 2.5 YBX1 promotes the translation of *RNF115* mRNA in an m5C‐dependent manner

**FIGURE 5:**
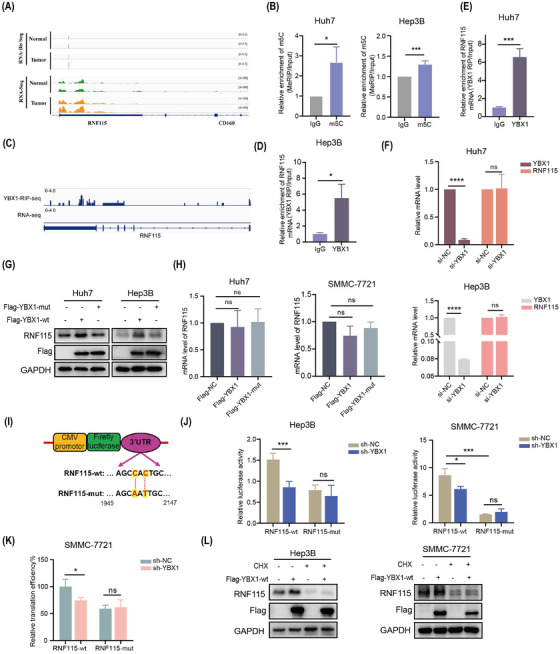
YBX1 promotes the translation of *RNF115* mRNA in a 5‐methylcytosine (m5C)‐dependent manner. (A) Genome Browser views of mRNA (RNA‐seq) and *RNF115* m5C‐mRNA (RNA‐Bis‐seq) signals in normal and tumour tissues in hepatocellular carcinoma (HCC). (B) Methylated RNA immunoprecipitation (MeRIP)‐quantitative polymerase chain reaction (qPCR) was used to detect the m5C modification level of *RNF115* mRNA in Huh7 and Hep3B cells, IgG was used as a control. (C) Genome Browser views of YBX1 binding signals for *RNF115* mRNA by YBX1 RNA immunoprecipitation (RIP)‐seq data. (D, E) RIP‐qPCR was used to detect the interaction between YBX1 and *RNF115* mRNA in Huh7 and Hep3B cells, and IgG was used as a control. (F–H) Knockdown of YBX1 (F) or overexpression of YBX1‐wt/YBX1‐mut (G, H), followed by detection of YBX1 protein and mRNA expression using western blotting (WB) and quantitative real‐time PCR (qRT‐PCR). (I) *RNF115* 3′‐untranslated region (UTR) containing either wild‐type or mutant (C‐to‐A/T mutation) m5C sites was cloned into luciferase reporter vector. (J, K) Relative luciferase activity (J) and translation efficiency (K) of the wild‐type and mutant forms of *RNF115* 3′‐UTR reporter vectors in Hep3B and SMMC‐7721 cells stably transfected with sh‐NC or sh‐YBX1, respectively. (L) Ring Finger Protein 115 (RNF115) expression was measured in Flag‐NC or Flag‐YBX1‐wt Hep3B and SMMC‐7721 cells with or without cycloheximide (CHX, 20 µM for 8 h) treatment. Data are shown as mean ± standard deviation (SD), *p < .05, ***p < .001, ****p < .0001, ns, not significant. Unpaired *t*‐test was used unless otherwise stated.

The findings of this study suggested that RNF115 is a potential target of YBX1 translation control. We further examined the role of m5C modification in YBX1‐mediated translation regulation. RNA‐seq and RNA‐Bis‐seq analysis showed that *RNF115* mRNA and its m5C modification level were elevated in HCC tumours, with the m5C modification site located in the 3′‐UTR, as confirmed by methylated RNA immunoprecipitation (MeRIP)‐quantitative polymerase chain reaction (qPCR; Figures 5A, B and S9A). RNA immunoprecipitation (RIP)‐seq data analysis revealed that YBX1 was enriched in the 3′‐UTR of *RNF115* mRNA, which was confirmed using RIP‐qPCR (Figures 5C–E and S9B). However, YBX1 knockdown significantly downregulated RNF115 protein expression without affecting its mRNA level, suggesting that YBX1 knockdown inhibited *RNF115* mRNA translation rather than its transcription (Figures 3I, S4C, D, 5F and S9C, D). The overexpression of YBX1‐wt, but not that of YBX1‐mut, significantly upregulated the protein expression of RNF115 (Figures 5G, H and S9E). To further explore the role of m5C modification in YBX1‐mediated *RNF115* mRNA translation, we conducted dual‐luciferase reporter assay. YBX1 knockdown downregulated the luciferase activity of overexpressed RNF115‐wt. However, overexpression of RNF115‐mut abolished the increase in luciferase activity induced by YBX1 (Figure 5I, J). Additionally, YBX1 knockdown significantly inhibited the translation efficiency of RNF115‐wt, whereas RNF115‐mut abolished this effect (Figure 5K). Furthermore, CHX completely reversed the YBX1‐wt overexpression‐induced upregulation of RNF115 (Figure 5L). These findings indicate that YBX1 mediates *RNF115* mRNA translation in an m5C‐dependent manner.

### 2.6 YBX1 interacts with EIF4A1 to promote *RNF115* mRNA circularisation

**FIGURE 6:**
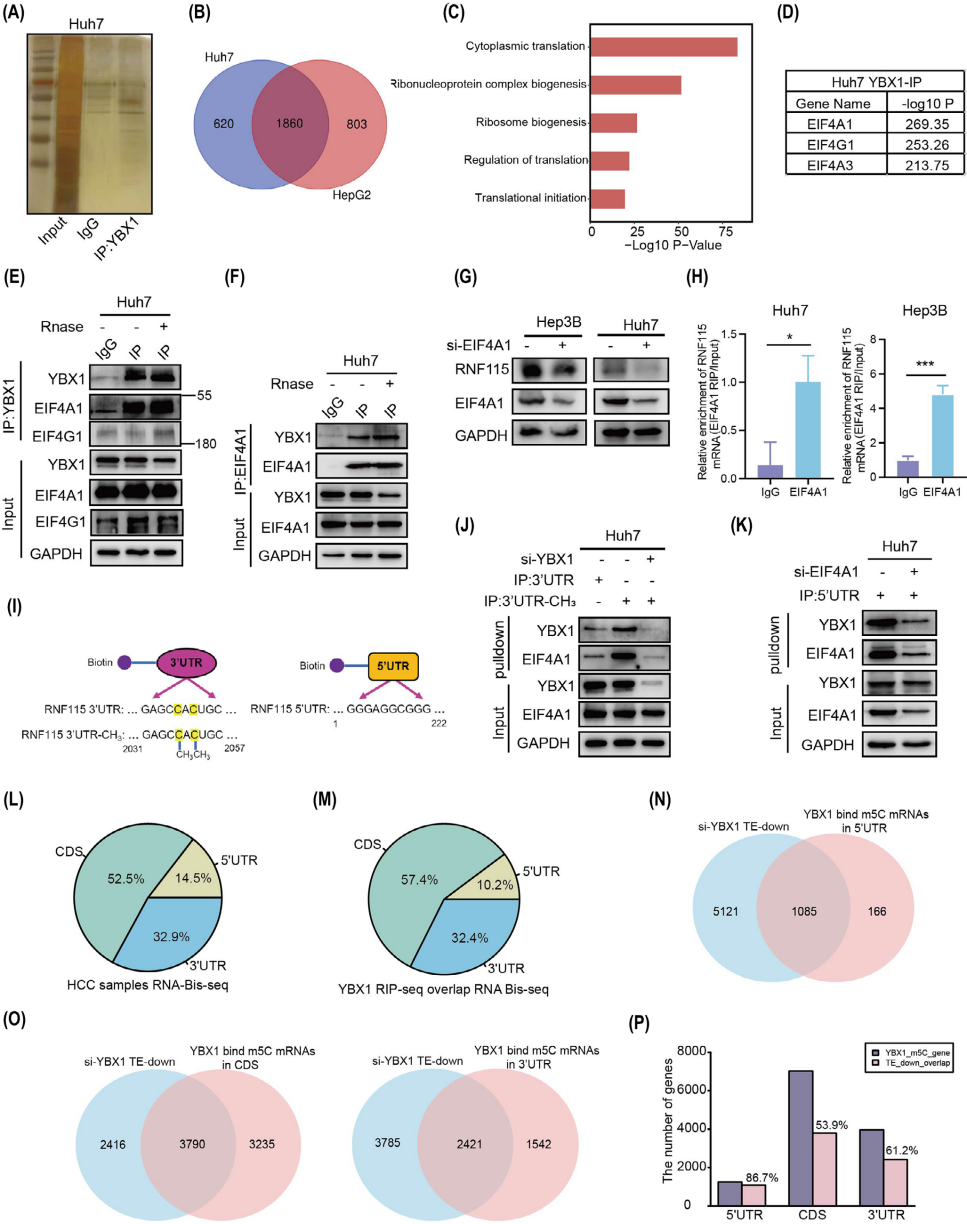
YBX1 interacts with Eukaryotic Translation Initiation Factor 4A1 (EIF4A1) to promote *RNF115* mRNA circularisation. (A) Images of Huh7 cells subjected to immunoprecipitation (IP) silver staining with anti‐YBX1 and IgG antibodies. (B, C) Venn diagram and Kyoto Encyclopedia of Genes and Genomes (KEGG) analysis showed the co‐interacting proteins with YBX1 in Huh7 and HepG2 cells. (D) Translation initiation‐associated proteins interacting with YBX1 were identified by IP MS/MS. (E, F) Co‐IP analysis was performed with or without Rnase (10 µg/mL, 37°C, 10 min) treatment in Huh7 cells, IgG as a control. (G) Knockdown of EIF4A1 in hepatocellular carcinoma (HCC) cells by si‐RNA after 48 h, Ring Finger Protein 115 (RNF115) expression was detected by western blotting (WB). (H) RNA immunoprecipitation (RIP) assay was performed to detect the interaction between EIF4A1 and *RNF115* mRNA, and IgG as a control. (I) Models for biotin‐labelled *RNF115* 3′‐untranslated region (UTR), 3′‐UTR‐CH_3_ and 5′‐UTR RNA. (J, K) RNA‐pulldown was performed to detect the interaction between YBX1, EIF4A1 and *RNF115* 3′‐UTR or 5′‐UTR after YBX1 or EIF4A1 knockdown in Huh7 cells. (L) Venn diagram showing the proportion of 5‐methylcytosine (m5C) modifications present in the coding sequence (CDS), 5′‐UTR and 3′‐UTR. (M) Venn diagram showing the distribution ratio of overlap genes between YBX1 RIP‐seq and m5C Bis‐seq in CDS, 5′‐UTR and 3′‐UTR. (N, O) Venn diagram showing the overlap of the downregulated translation efficiency genes after YBX1 knockdown with the YBX1‐binding 5′‐UTR (N), CDS (O, left panel) and 3′‐UTR (O, right panel) m5C‐modified mRNAs. (P) Histograms and distribution ratio of YBX1‐bound m5C‐modified mRNAs and genes with downregulated translation efficiency after YBX1 knockdown. Data are shown as mean ± standard deviation (SD), *p < .05, ***p < .001. Unpaired *t*‐test was used unless otherwise stated.

To investigate the mechanism by which YBX1 promotes RNF115 mRNA translation in HCC cells, Co‐IP assay and MS/MS analysis were performed. We identified that YBX1 interacted with 1860 proteins, which were closely associated with the regulation of translation (Figure 6A–C). Analysis of the pathways involved in translation initiation revealed that YBX1 mainly interacted with EIF4A1, EIF4A3, EIF4G1 and so forth (Figure 6D). Due to the high abundance of EIF4A3 in the nucleus and the weak interaction between YBX1 and EIF4G1, we speculated that EIF4A1 played a pivotal role in translation initiation. As expected, the interaction of YBX1 and EIF4A1 was strong and RNA‐independent (Figures 6E, F and S9F, G). Next, we examined whether YBX1 promoted *RNF115* mRNA translation by regulating EIF4A1 expression. Interestingly, neither YBX1 knockdown nor overexpression affected EIF4A1 expression (Figure S9H, I). However, EIF4A1 knockdown significantly downregulated the expression of RNF115 (Figures 6G and S9J). The interaction between EIF4A1 and *RNF115* mRNA was confirmed using the RIP assay (Figure 6H). To elucidate how YBX1 regulates translation in an m5C‐dependent manner, we performed RNA pulldown and revealed that YBX1 and EIF4A1 preferentially bound to *RNF115* m5C modification sites in the 3′‐UTR. Moreover, YBX1 knockdown significantly inhibited the binding of EIF4A1 to 3′‐UTR‐CH_3_ in SMMC‐7721 and Huh7 cells (Figures 6I,J and S9K). These data suggested that EIF4A1 bound to *RNF115* 3′‐UTR‐CH_3_ through interaction with YBX1. Similarly, the 5′‐UTR pulldown of *RNF115* mRNA showed that EIF4A1 and YBX1 bound to 5′‐UTR, and the binding of YBX1 to 5′‐UTR was abolished after EIF4A1 knockdown (Figures 6K and S9L). These results support a model that YBX1 preferentially binds to the m5C modification sites of *RNF115* 3′‐UTR and interacts with EIF4A1 to bridge *RNF115* 5′‐UTR, thereby promoting mRNA circularisation.

Notably, YBX1 regulates global protein synthesis in an m5C‐dependent manner (Figure 3F). We further examined the impact of YBX1 on the translation efficiency of other existing m5C‐modified mRNAs. Analysis combining YBX1 RIP‐seq and m5C Bis‐seq data revealed that 12 239 gene mRNAs with m5C modification were bound by YBX1, including *RNF115*, which were mainly distributed in coding sequence (CDS; 57.4%, 7025/12 239) and 3′‐UTR (32.4%, 3963/12 239) regions (Figure 6L, M). YBX1 knockdown decreased the translation efficiency of genes with m5C sites deposited in the 5′‐UTR (86.7%, 1085/1251), CDS (53.9%, 3790/7025) and 3′‐UTR (61.2%, 2421/3963), respectively (Figure 6N‐P). These results suggested that YBX1 can bind to transcripts with m5C modification (such as *RNF115* mRNA with m5C sites at the 3′‐UTR) and EIF4A1 to promote mRNA translation.

### 2.7 NSUN2 is the m5C methyltransferase of *RNF115* mRNA to inhibit ferroptosis

**FIGURE 7:**
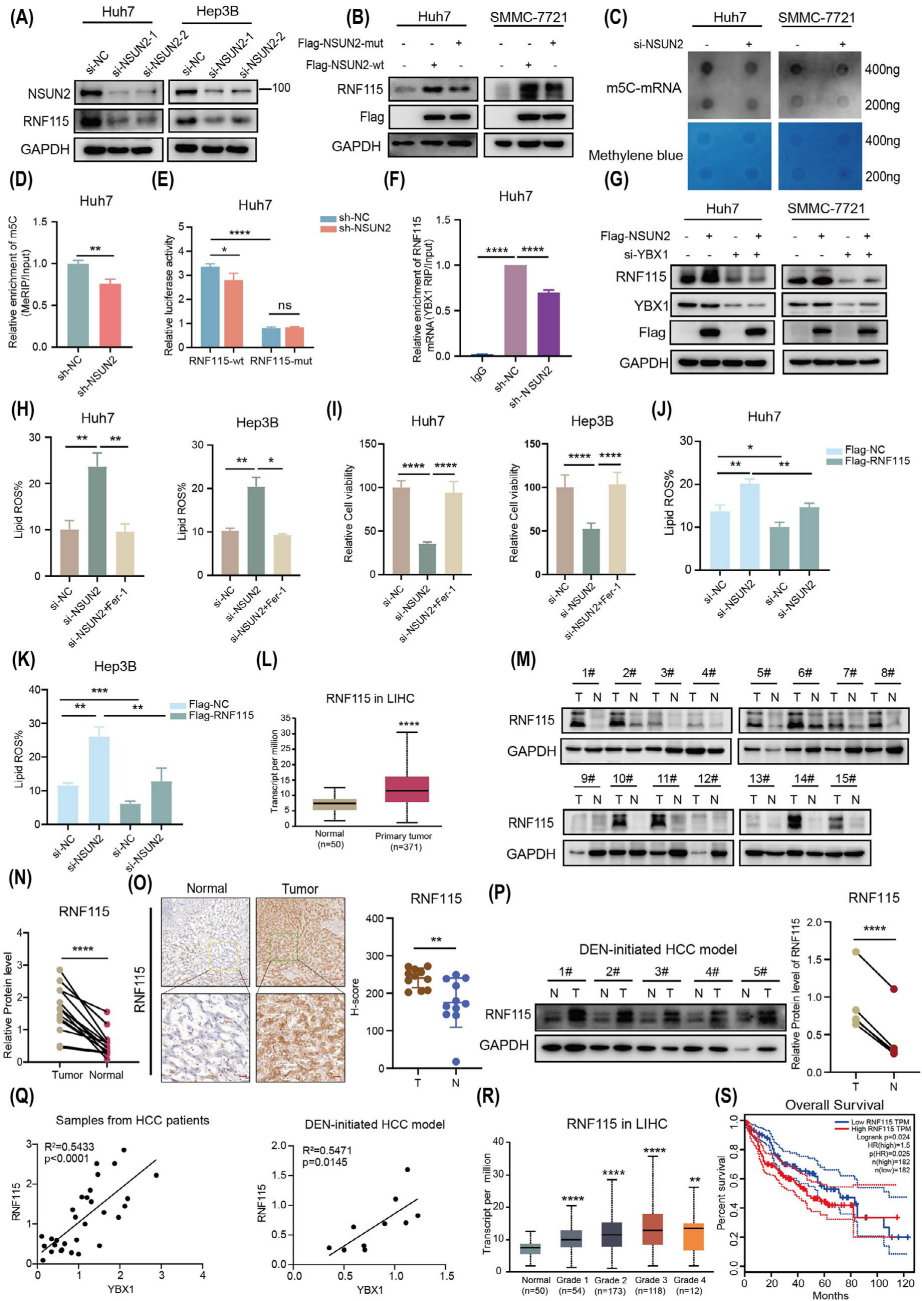
NOP2/Sun RNA methyltransferase 2 (NSUN2) is the 5‐methylcytosine (m5C) methyltransferase of *RNF115* mRNA to inhibit ferroptosis. (A) Knockdown of NSUN2 in Huh7 and Hep3B cells by si‐RNA after 48 h, Ring Finger Protein 115 (RNF115) expression was detected by western blotting (WB). (B) WB analysis was used to detect RNF115 expression after stable transfection of Flag‐NSUN2‐wt and Flag‐NSUN2‐mut in Huh7 and SMMC‐7721 cells. (C, D) The m5C modification level of total mRNA (C) or *RNF115* mRNA (D) after NSUN2 knockdown was detected by dot blot or Methylated RNA immunoprecipitation (MeRIP)‐quantitative polymerase chain reaction (qPCR). (E) Relative luciferase activity of the wild‐type and mutant forms of RNF115 3′‐untranslated region (UTR) reporter vectors in Huh7 cells stably transfected with sh‐NC or sh‐NSUN2, respectively. (F) After knockdown YBX1, the binding level of YBX1 and *RNF115* mRNA was detected by RNA immunoprecipitation (RIP)‐qPCR. (G) After overexpression of NSUN2 combined with knockdown of YBX1 in SMMC‐7721 and Huh7 cells, the expression level of RNF115 was detected by WB. (H, I) NSUN2 knockdown combined with Fer‐1 treatment was used to detect lipid reactive oxygen species (ROS) level (Fer‐1: 1 µM, 8 h) and cell viability (Fer‐1: 780 nM for 72 h). (J, K) Lipid ROS levels were detected by knockdown NSUN2 in Huh7 and Hep3B cells with stable expression of Flag‐RNF115. (L) RNF115 expression levels were analysed by The Cancer Genome Atlas (TCGA) database. (M, N) Western blot was used to analyse the expression of RNF115 in 15 pairs of hepatocellular carcinoma (HCC) tissue samples and statistical analysis (****p < .0001, paired *t*‐test. T, tumour. N, normal). (O) RNF115 immunohistochemical (IHC) staining and statistical analysis (T, tumour = 11, N, normal = 11, unpaired *t*‐test). Scale bar: 100 µm (upper panel) and 50 µm (lower panel). (P) WB analysis was used to detect RNF115 expression in diethylnitrosamine (DEN)‐initiated HCC model (****p < .0001, paired *t*‐test. T, tumour. N, normal). (Q) The correlation analysis of YBX1 and RNF115 protein expression in HCC patient samples and DEN‐initiated HCC mouse samples. (R) RNF115 expression levels in different grades of progress by TCGA database analysis. (S) Correlation analysis of YBX1 expression levels with overall survival using GEPIA database (http://gepia.cancer‐pku.cn/). Data are shown as mean ± standard deviation (SD), *p < .05, **p < .01, ***p < .001, ****p < .0001, ns, not signification. Unpaired *t*‐test was used unless otherwise stated.

The findings of this study demonstrated that YBX1 inhibited ferroptosis by facilitating the translation of *RNF115* mRNA in an m5C‐dependent manner. Therefore, it was imperative to identify the methyltransferase responsible for mediating the m5C modification of *RNF115* mRNA. We preformed correlation analysis between methyltransferases known to mediate mRNA m5C modification, including NSUN2, DNMT2, NSUN6 and so forth and the RNF115 transcript. NSUN2 exhibited the highest correlation with RNF115 (*R* = .43, *p* = 0; Figure S10A, B). Two si‐RNA constructs specifically targeting NSUN2 significantly downregulated RNF115 expression (Figures 7A and S10C). As both si‐RNA constructs exhibited similar NSUN2 knockdown efficiency, we used si‐NSUN2‐2 in all subsequent experiments (referred to as si‐NSUN2 hereafter). However, overexpression of NSUN2‐wt, but not NSUN2‐mut (C271/321A, mutant of NSUN2 methyltransferase activity), promoted the expression of RNF115 (Figure 7B). Moreover, NSUN2 knockdown reduced the global mRNA m5C modification level as well as the m5C modification level of *RNF115* mRNA (Figures 7C, D and S10D, E). Furthermore, NSUN2 knockdown significantly inhibited the luciferase activity of overexpressed RNF115‐wt, while overexpressing RNF115‐mut abolished the regulatory effect of NSUN2 (Figures 7E and S10F). It is noteworthy that NSUN2 knockdown inhibited the interaction between YBX1 and *RNF115* mRNA (Figures 7F and S10G). In addition, YBX1 knockdown abolished the promotion of RNF115 expression by overexpressing NSUN2 (Figure 7G). This suggested that the regulation of RNF115 by NSUN2 is dependent on YBX1. To further explore the role of NSUN2 in ferroptosis, NSUN2 knockdown inhibited the proliferative capacity and mitochondrial membrane potential of Hep3B, Huh7 and SMMC‐7721 cells, while promoting the accumulation of lipid ROS. However, Fer‐1 completely reversed this effect (Figures 7H, I and S10H–J). Overexpression of NSUN2‐wt, but not that of NSUN2‐mut, significantly promoted the proliferation of HCC cells (Figure S10K, L). Moreover, the overexpression of RNF115 reversed the NSUN2 knockdown‐induced accumulation of lipid ROS (Figure 7J, K). These results suggest that NSUN2 inhibits ferroptosis by mediating m5C modification of *RNF115* mRNA.

To evaluate the clinical importance of RNF115 in HCC, the expression levels of RNF115 were analysed in TCGA datasets. RNF115 expression in HCC was elevated compared to normal tissues (Figure 7L). Consistently, WB, IHC and DEN‐initiated HCC model analysis also revealed that RNF115 was elevated in HCC (Figure 7M–P), and in both HCC patient samples and the DEN‐initiated HCC model, the protein expression of RNF115 was significantly positively correlated with YBX1 (Figure 7Q). Moreover, RNF115 expression was progressively upregulated with HCC progression (Figure 7R). The upregulated expression of RNF115 was correlated with decreased overall survival (OS) in patients (Figure 7S). These findings indicate that RNF115 is crucial for HCC development.

## 3 DISCUSSION

**FIGURE 8:**
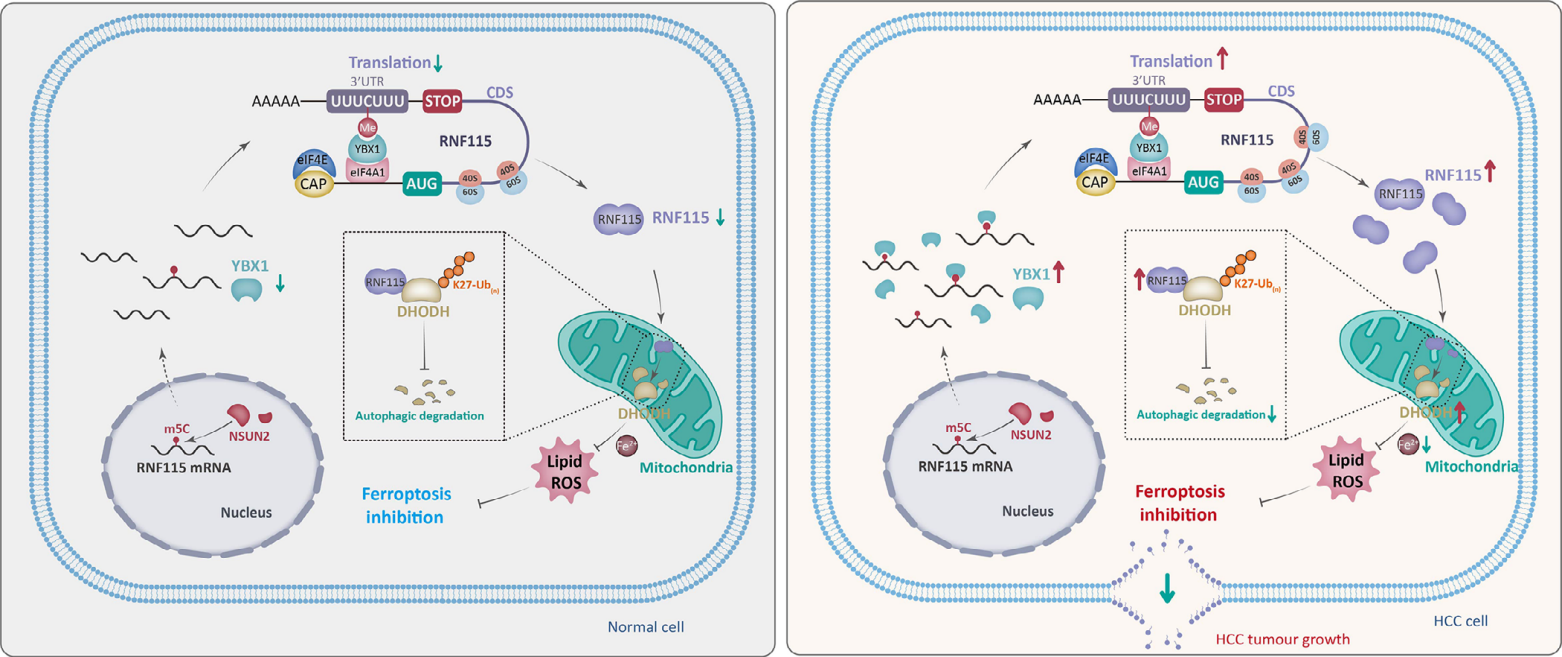
YBX1/NOP2/Sun RNA methyltransferase 2 (NSUN2) promotes hepatocellular carcinoma (HCC) progression by inhibiting ferroptosis through Ring Finger Protein 115 (RNF115)–dihydroorotate dehydrogenase (DHODH) axis. NSUN2 promotes 5‐methylcytosine (m5C) modification of *RNF115* mRNA, which is subsequently bound by YBX1. YBX1 interacts with Eukaryotic Translation Initiation Factor 4A1 (EIF4A1) to promote *RNF115* mRNA looping and translation, thereby enhancing RNF115 expression. Subsequently, RNF115 interacts with DHODH to promote its K27 ubiquitination and inhibit the autophagic degradation of DHODH to resist ferroptosis and promote HCC progression.

Ferroptosis is suppressed in a variety of tumours, including HCC, and indicating ferroptosis may provide a promising strategy for cancer treatment.^36^, ^37^ A few reports have shown that RNA binding proteins are important in the regulation of ferroptosis.^38^, ^39^ YBX1 plays an important role in HCC, including tumour progression, sorafenib resistance, anti‐tumour immune response and so forth.^40^, ^41^, ^42^ However, the role of YBX1 as an mRNA m5C binding protein in ferroptosis remains unclear. This study demonstrated that YBX1 inhibited ferroptosis through the RNF115–DHODH signalling axis in an m5C‐dependent manner (Figure 8).

In this study, we performed LC–MS/MS proteomics analysis by knocking down YBX1 in Huh7 cells and found that the ferroptosis signalling pathway was significantly enriched, suggesting that YBX1 may mediate the regulation of ferroptosis signalling. *PTGS2*, which encodes the COX2 protein, is considered a marker gene for ferroptosis.^27^, ^28^, ^29^, ^30^ Therefore, we evaluated the activation of ferroptosis by the expression level of *PTGS2*. In the DEN‐initiated HCC model, *PTGS2* expression was significantly suppressed in tumours, indicating that ferroptosis is suppressed in HCC, and activation of ferroptosis might be a potential strategy to inhibit HCC progression. Based on this, we observed that knockdown of YBX1 significantly upregulated the expression of *PTGS2* mRNA and COX2 protein, further confirming the role of YBX1 in the resistance to ferroptosis in HCC.

Our study shows that RNF115 is localised in mitochondria and inhibited ferroptosis by promoting DHODH expression. DHODH is mainly located in mitochondria and uses Coenzyme Q10 (CoQ10) to resist abnormal oxidation state in cells and inhibit oxidative stress response.^33^, ^43^ RNF115, an E3 ubiquitin ligase, regulates the ubiquitin modification of various substrates and is essential for the progression of tumours.^44^, ^45^, ^46^ However, the precise mechanism by which RNF115 acts as an E3 ubiquitin ligase to regulate DHODH expression has yet to be fully understood. We discovered that RNF115 strongly interacted with DHODH and promoted K27 ubiquitination, inhibiting its autophagic degradation to resist ferroptosis. Moreover, GPX4 is an important protein in the resistance to ferroptosis. We found that knockdown of RNF115 induced autophagy but did not affect GPX4 expression, suggesting that GPX4 is not degraded through autophagy. Studies have shown that USP8 can remove the ubiquitination modification of GPX4, stabilising it,^27^ which further confirms that GPX4 is not degraded via autophagy.

There is no doubt that ferroptosis holds great potential in cancer therapy. However, its activation has been reported to cause liver damage and renal toxicity.^40^, ^41^ Additionally, the activation of ferroptosis signalling in CD8^+^ T cells has been shown to lead to resistance to PD1 monoclonal antibody therapy.^47^ Therefore, ferroptosis remains a double‐edged sword in disease treatment, offering both therapeutic promise and potential risks.

The mRNA translation program is essential for determining tumour fate. Previous research has demonstrated that targeting protein translation is a promising therapeutic strategy for cancer.^31^ It is worth emphasising here that the level of m5C modification and mRNA translation are interdependent.^8^ Meanwhile, m5C modification regulates the translation of target mRNAs.^11^, ^48^ However, the exact mechanism through which m5C modification regulates translation is unclear. YBX1 has been identified as a DNA and RNA binding protein, transcription factor and m5C reader, promoting the progression of various tumours by regulating transcription and mRNA stability. Additionally, YBX1 has been reported to regulate target mRNAs translation by recruitment to polysomal chains,^16^ but the specific role of m5C modification in this processing is still unknown. In this study, YBX1 strongly interacted with EIF4A1 to promote RNF115 expression. EIF4A1 has previously been reported to directly bind to purine‐rich regions of mRNA 5′‐UTR to promote mRNA translation.^49^ Further research demonstrated that YBX1 did not affect the expression of EIF4A1, but instead recognised and bound to the m5C modification sites of *RNF115* 3′‐UTR interacting with EIF4A1 to bridge the 5′‐UTR, thereby promoting *RNF115* mRNA circularisation and translation. RNA Bis‐seq analysis revealed that m5C is predominantly enriched in the 3′‐UTR and CDS. Meanwhile, RIP‐seq analysis revealed a high overlap between YBX1 and the m5C‐modified regions. YBX1 recognised the m5C modification sites in the 3′‐UTR and interacted with EIF4A1 to promote mRNA circularisation and translation. Ribo‐seq analysis demonstrated that most of the mRNAs recognised by YBX1 are regulated at the translational level. This finding enriches the understanding of YBX1's novel functions and highlights the important role of the translation process in HCC progression. However, additional research is required to determine the presence of m5C modifications in the CDS and 5′‐UTR and if these findings align with the translation model. Nevertheless, our results have similarities with the regulation of mRNA translation by N6‐methyladenosine (m6A) modification mediated by methyltransferase 3 (METTL3), which promoted mRNA circularisation and enhanced translation by interacting with Eukaryotic Translation Initiation Factor 3 Subunit H (EIF3h).^50^ In conclusion, this study elucidated a specific mechanism underlying the m5C modification‐mediated regulation of translation, at least in *RNF115* mRNA.

Targeting RNA hypermethylation is a promising clinical therapeutic strategy. The small‐molecule inhibitor STC‐15, targeting the m6A methyltransferase METTL3, has entered clinical trials (ClinicalTrials.gov ID: NCT05584111) and demonstrated promising therapeutic effects against solid tumours. With further research, highly specific and potent small‐molecule compounds are gradually being identified, demonstrating promising inhibitory effects on haematologic malignancies and other diseases.^51^, ^52^ However, small‐molecule inhibitors targeting m5C methylation are still in the early stages of development. Recently, several small‐molecule inhibitors targeting the m5C methyltransferase NSUN2 have been identified, showing promising anti‐tumour effects.^53^, ^54^ Meanwhile, the small‐molecule inhibitor SU056, targeting YBX1, has shown encouraging therapeutic effects in treating triple‐negative breast cancer in preclinical studies.^31^ These findings highlight the great potential of targeting m5C methylation in disease treatment. However, more efforts are needed in the search for specific small‐molecule inhibitors.

The upregulated expression of YBX1 in multiple tumours correlates with poor prognosis.^14^, ^55^, ^56^ In this study, knockdown of YBX1, especially when combined with the ferroptosis inducer FIN56, significantly reduced the tumour volume without affecting the body weight of the xenograft mice. The knockdown of RNF115, a downstream target of YBX1, mitigated the inhibitory effect of YBX1 on ferroptosis. In addition, analysis of clinical samples revealed that YBX1 and RNF115 were upregulated in HCC tumours and associated with poor prognosis. Therefore, targeting the YBX1–RNF115 axis‐mediated ferroptosis may be beneficial for HCC treatment.

In summary, we found that YBX1 promoted the progression of HCC by inhibiting ferroptosis through facilitating *RNF115* mRNA circularisation and translation in an m5C‐dependent manner. Moreover, RNF115 was identified as a novel identified E3 ubiquitin ligase for DHODH, mediating its K27 ubiquitination and autophagic degradation. YBX1 recognised and bound to m5C modification sites and interacted with EIF4A1, facilitating *RNF115* mRNA circularisation and translation, which in turn promoted DHODH expression. Meanwhile, NSUN2 was responsible for the m5C modification of *RNF115* mRNA, inhibiting ferroptosis. Abolition of the YBX1–RNF115–DHODH axis may be a potential therapeutic strategy for HCC by ferroptosis.

## 4 METHODS

### 4.1 Cell culture and reagents

Hep3B, Huh7, SMMC‐7721, HepG2 and HEK293T were purchased from Cell Bank of the Chinese Academy of Sciences and validated via short tandem repeat (STR) analysis. The cells were cultured in DMEM (Gibco) supplemented with 10% foetal bovine serum (FBS, Invigentech) and maintained at 37°C with 5% CO_2_. FIN56 (GC30039), CQ (GC19549), 3‐MA (GC10710), RSL3 (GC12431), erastin (GC16630), Fer‐1 (GC16630), CHX (GC17198), MG132 (GC10383) and BQR (GC19082) were obtained from Glpbio and dissolved to the specified concentration in DMSO or H_2_O.

### 4.2 Patient specimens

Patient specimens were obtained from the Biobank of the First Affiliated Hospital of Zhengzhou University. The present study received approval from the Ethics Committee of Scientific Research and Clinical Trial of the First Affiliated Hospital of Zhengzhou University (Approval 2022‐KY‐0005‐001), and adhered to the Declaration of Helsinki. Written informed consent was obtained from all participants in accordance with institutional guidelines.

### 4.3 Cell viability assay

Seed cells at a concentration of 3000 per well in a 96‐well plate. After exposure to the corresponding compound or si‐RNA for the designated time, each well was treated with 10 µL of Cell Counting Kit‐8 (CCK8) reagent (K1018, APExBIO). Following 1.5 h incubation at 37°C, absorbance at 450 nm was recorded using a microplate reader (Perkin Elmer).

### 4.4 Analysis of lipid ROS production

Seed cells at a concentration of 2.5 × 10^5^ per well in six‐well plates. After 24 h, the cells were treated with the specified compounds for the designated durations and collected using an EDTA‐free trypsin digest (Beyotime), followed by phosphate‐buffered saline (PBS) washing, after which the cells were treated with 2 µM C11‐bodipy (581/591; diluted in PBS; GC40165, Glpbio) at 37°C for 30 min. Subsequently, the cells were rinsed with PBS, resuspended in 500 µL of PBS, and assayed using a flow cytometer (BD Biosciences).

### 4.5 Quantitative real‐time PCR

Total RNA was isolated with TRIzol reagent (Invitrogen) and converted into complementary DNA (cDNA) through reverse transcription, utilising PrimeScript™ RT reagent Kit with gDNA Eraser (Takara). Quantitative real‐time PCR (qRT‐PCR) was performed by the TB Green^®^ *Premix Ex Taq*™ II (Tli RNaseH Plus; Takara) in compliance with the manufacturer's protocol. Glyceraldehyde 3‐phosphate dehydrogenase (GAPDH) used as an endogenous control, and the data were expressed as relative fold changes. The primer sequences utilised for qPCR analysis are listed in Table S1.

### 4.6 Western blotting analysis

Protein was extracted with Radio Immunoprecipitation Assay (RIPA) lysis buffer (P0013B, Beyotime) containing Phenylmethylsulfonyl fluorid (PMSF) (Beyotime), phosphatase inhibitor cocktail (Roche) and protease inhibitors (K0010, MCE). The protein concentration was determined using a BCA Protein Assay Kit (Beyotime). Twenty micrograms of total protein lysates were resolved via sodium dodecyl sulphate‐polyacrylamide gel electrophoresis (SDS‐PAGE) gel electrophoresis and analysed using detection antibodies. The primary antibodies utilised in this study are detailed below: anti‐YBX1 (abcam, ab76149), anti‐RNF115 (abcam, ab187642), anti‐GPX4 (abcam, ab125066), anti‐DHODH (abcam, ab174288), anti‐GAPDH (abcam, ab8245), anti‐NSUN2 (Proteintech, 20854‐1‐AP), anti‐LC3 (Proteintech, 14600‐1‐AP), anti‐P62 (Proteintech, 18420‐1‐AP), anti‐His (Proteintech, 66005‐1‐Ig), anti‐HA (Proteintech, 66006‐2‐Ig), anti‐Flag (CST, 14793), anti‐Ub (Proteintech, 10201‐2‐AP), anti‐EIF4G1 (abcam, ab2609), anti‐EIF4A1 (abcam, ab185946), anti‐IgG (Proteintech, 3000‐0‐AP), anti‐FSP1 (Proteintech, 20886‐1‐AP), anti‐ACSL3 (20710‐1‐AP), anti‐SLC7A11 (26864‐1‐AP), anti‐ZNF669 (Abcepta, AP58167).

### 4.7 Immunohistochemistry (IHC) staining

Briefly, tissue samples were preserved with formalin and embedded in paraffin blocks. Using a microtome, 5 µm sections were cut from paraffin blocks, followed by deparaffinisation and dehydration. Antigen retrieval was performed with either EDTA or citrate buffer, followed by blocking with 5% goat serum. Primary antibodies targeting YBX1 (abcam, ab76149), RNF115 (Sigma, HPA019130), DHODH (Proteintech, 14877‐1‐AP), 4‐HNE (abcam, ab48506) and Ki67 (GB111141‐100, Servicebio) were applied to tissue sections. Subsequently, the secondary antibody was used for incubation at 37°C for 2 h, followed by staining using a DAB kit. The images were captured using the 3DHISTECH PANNORAMIC VIEWER.

### 4.8 IHC H‐score analysis

In brief, according to the 3DHISTECH QuantCenter 2.2 User Guide for IHC quantification analysis, the process begins with using PatternQuant to train the target tissue, establishing a segmentation model and automatically segmenting the regions of interest (such as tissues or cell structures). Then, based on the target protein, analysis modes such as NuclearQuant, MembraneQuant or CellQuant are selected. A quantification template is created by defining the proportions of strong positive, moderate positive, weak positive and negative staining, followed by quantification scoring for each target tissue.

### 4.9 Immunofluorescence staining

The cells were fixed paraformaldehyde for 20 min at 25°C, then rinsed with PBS, permeabilisation with .02% Triton‐100 for 15 min, washing with PBS again and blocking with 5% BSA for 20 min. Subsequently, the samples were incubated with primary antibodies overnight at 4°C, followed by a 2 h incubation with fluorescent secondary antibodies at room temperature and imaging was performed using confocal microscopy (Zeiss, LSM900). The primary antibodies employed in IF were: anti‐RNF115 (Sigma, HPA019130), anti‐VDAC3 (Proteintech, 55260).

### 4.10 Co‐immunoprecipitation and mass spectrometry

Briefly, 10 µg antibody was cross‐linked to protein A/G by co‐IP cross‐linking kit (88805, Thermo). Cells were lysed using IP lysis buffer, and 1000 µg of total protein was incubated overnight at 4°C with an antibody‐protein A/G complex. After incubation, the mixture was washed five times using IP lysis and eluted with elution buffer. The eluted samples were combined with 1× loading buffer and heated at 100°C for 10 min. Where indicated, the affinity elute was processed to SDS‐PAGE, followed by either colloidal silver staining or WB. Bands were excised and analysed by mass spectrometric for sequencing (4D Label free/DIA, APExBIO).

### 4.11 Ferrous iron measurement

Intracellular ferrous iron was measured by labelling cells with FerroOrange (F374, Dojindo) at 1 µM for 30 min in PBS at 37°C and analysed by flow cytometry.

### 4.12 Measurement of mitochondrial membrane potential

In brief, following the steps of the mitochondrial membrane potential detection kit (C2006, Beyotime). First, collect 200 000 cells and wash once with PBS. Combine 500 µL of complete medium with 500 µL of 1X JC‐1 working solution and incubate at 37°C for 20 min. After centrifuging at 1000 rpm and washing with PBS, add 500 µL of working solution and proceed with analysis using a BD flow cytometer.

### 4.13 RNA interference and plasmid transfection

All plasmids and si‐RNAs were diluted in Opti‐MEM (Gibco) and transfected into the cell lines using Lipofectamine RNAiMAX or Lipofectamine 3000 (Invitrogen) in compliance with the manufacturer's recommended protocol. The sequences targeted by the si‐RNA, sgRNA or shRNA are listed in Table S2.

The plasmids, sg‐YBX1, sh‐YBX1, sh‐NSUN2, sh‐RNF115, 3FLAG‐YBX1‐wt, 3FLAG‐YBX1‐mut (W65A), HA‐Ub (K6, K11, K27, K29, K33, K48, K63) were obtained from GENECHEM. HA‐K27R, His‐DHODH, 3FLAG‐RNF115, 3FLAG‐RNF115‐2CA were purchased from Tsingke.

### 4.14 Stably overexpressed/depleted cell lines construction

For YBX1, RNF115 and NSUN2 stable overexpression or knockdown cell lines were constructed, the corresponding viral plasmids (GENECHEM) were co‐transfected with pMD2.G vector and PsPAX2 vector into HEK293T cells by Lipofectamine 3000 (Invitrogen) for 24 h. The supernatant was separated, concentrated and filtered. Cells were infected with the filtered medium for 24 h, and cells expressing the target were selected using puromycin (2 µg/mL, Solarbio, P8230) or Hygromycin B (500 µg/mL, Glpbio, GC15496) and then verified by WB or qRT‐PCR.

### 4.15 Puromycin intake assay

Protein synthesis was detected using puromycin antibody (MABE343, Millipore, 1:20 000 dilution). Following treatment, cells were exposed to 1 µM puromycin (Solarbio, P8230) for 1 h, then harvested. Proteins were extracted for WB analysis, with GAPDH used as an endogenous control.

### 4.16 Dual‐luciferase reporter assay and translation efficiency

Luciferase reporter plasmids carrying either RNF115‐wt or RNF115‐mut sequences were constructed by GENECHEM and inserted into GV272 vector. After 24 h of plating cells in 96‐well plates, each well was transfected with 200 ng of luciferase reporter plasmid and 10 ng of Renilla plasmid. After 24 h, the luciferase activity was measured by the dual‐luciferase reporter assay kit (E1910, Promega). Translation efficiency using the previously described method.^50^

### 4.17 RNA immunoprecipitation

Cells were harvested, lysed using RIP lysis Buffer (Protease Inhibitor, 10 mM HEPES pH 7.6, 150 mM KCl, .5% NP‐40, 2 mM EDTA, .5 mM DTT, RNase Inhibitor) for 30 min at 4°C. Afterwards, the cell lysate was separated by centrifugation at 15 000 × *g* for 20 min for use. YBX1 or EIF4A1 antibody was incubated with Protein A magnetic beads for 1 h at room temperature, and then cell lysates were incubated with YBX1 or EIF4A1 antibody and Protein A magnetic beads for 4 h at 25°C. This was followed by elution with Protein K at 55°C. The eluted complexes were then subjected to RNA extraction for subsequent quantification and analysis via qRT‐PCR.

### 4.18 Methylated RNA immunoprecipitation

Cells were collected to extract total RNA and enriched for mRNA through mRNA enrichment kit (Invitrogen, 61006). The mRNA was subsequently interrupted into 100–200 bp fragments using the mRNA interruption reagent (Invitrogen, AM8740). The 3 µg m5C antibody (abcam, ab10805) was incubated with Protein A magnetic beads (Thermo, 88846) for 1 h, followed by incubation of 400 ng of mRNA fragments with them for 4 h at room temperature, after which RNA was eluted by Protein K at 55°C and extracted for qRT‐PCR.

### 4.19 Dot blot assay

In brief, cells were harvested, RNA was extracted and mRNA was enriched by mRNA Capture Beads (Vazyme, N401‐02). The nylon membrane (Solarbio, YA1760) was activated in 1 × Saline Sodium Citrate (SSC) buffer (Sigma, S6639) for 2 h and dried at 37°C. A total of 200 or 400 ng of mRNA was UV‐linked to a nylon membrane, blocked with milk for 1 h, incubated with m5C antibody (abcam, ab10805) overnight, and visualised after incubation with secondary antibody for 2 h.

### 4.20 Ribo‐seq

Cells at 70%–80% confluency were exposed to 100 µg/mL cycloheximide (C1988, Sigma‐Aldrich) for 10 min at 37°C to halt translation, then lysed in ice‐cold lysis buffer (150 mM NaCl, 20 mM Tris–HCl pH 7.4, 5 mM MgCl_2_, 100 µg/mL CHX, 1% Triton X‐100, 1 mM DTT; 25 U/mL Turbo DNase (AM2239, Ambion)) for 10 min on ice. The lysate was centrifuged at 20 000 × *g* for 10 min at 4°C to clarify. Subsequently, RNase I (100 U/µL, AM2295, Ambion) was applied, and the mixture was incubated at room temperature for 45 min with gentle agitation. Nuclease digestion was stopped by adding SUPERase In RNase inhibitor (AM2694, Invitrogen). The mixture was layered onto a 700 µL sucrose cushion (1 M sucrose) and centrifuged at 260 000 × *g* for 4 h at 4°C using an S140AT‐2545 rotor in Himac CS150NX ultracentrifuge. After removing the supernatant, the pellet was resuspended in 50 µL of pellet buffer (10 mM Tris pH 7.5 and 1% SDS). Foot printing RNA was isolated from the resuspension using 1 mL TRIzol (15596018, Life Technologies) and 200 µL chloroform. RNA samples were resolved on a 15% Tris‐Borate‐EDTA (TBE)–urea polyacrylamide (EC68852BOX, Invitrogen), and fragments ranging from 17 to 34 nucleotides were excised and collected. Next, RNA samples were extracted by using small‐RNA™ PAGE recovery kit (R1070, Zymo). Before constructing the library, RNA fragments underwent end‐repair with T4 polynucleotide kinase (M0201S, New England Biolabs). RNA was purified using the ZR oligo clean concentrator (D4061, Zymo). The sample was prepared for sequencing using the VAHTS™ small RNA library prep kit for Illumina (NR801, VAHTS). Barcode‐labelled libraries were combined and sequenced using Illumina sequencing platforms with 150 bp paired‐end reads.

### 4.21 RIP‐seq

Cells were rinsed twice with chilled PBS, extract RNA by using 1 mL TRIzol reagent (15596018, Invitrogen) and 200 µL chloroform. Adjust the volume of 75 µL to 100 µL DEPC‐treated water. Using large‐orifice tips or cut off the end of regular micropipette tips for transferring 40 µL protein A beads (10001D, Invitrogen). Rinse the beads three times with 1 mL Immunopreciptation Pre‐made (IPP) buffer (10 mM Tris (pH 7.4), 150 mM NaCl, .1% NP40) for 3 min with gentle rotation at 25°C, then resuspend the beads in 500 µL IPP buffer with RNaseOUT (10777‐019, Invitrogen). Mix 10 µg of YBX1 antibody with protein A beads and incubate for 1 h with gentle rotation. Fragmented RNA was added into beads–antibody mixture, and incubated for 4 h with gentle rotation at 4°C. RNA enrichment was normalised to IgG. For sequencing, rRNAs was eliminated using the NEBNext rRNA Depletion Kit (E7400L, New England BioLabs). Subsequently, the cDNA libraries were generated with the VAHTS universal V8 RNA‐seq library prep kit for Illumina (NR605, VAHTS) and sequenced on Illumina NovaSeq 6000 platform. Each group was analysed in duplicate.

### 4.22 RNA‐seq

RNA‐seq was performed following the protocol of the VAHTS universal V8 RNA‐seq library prep kit for Illumina (NR605, VAHTS). In brief, total RNA was extracted from cells using TRIzol reagent (15596018, Life Technologies). mRNA was then purified using VAHTS mRNA capture beads (N401‐02, VAHTS) and used to generate cDNA libraries. All samples were sequenced on Illumina NovaSeq 6000 platform.

### 4.23 RNA‐pulldown

Biotin‐tagged *RNF115* 3′‐UTR and 3′‐UTR‐CH_3_ were synthesised in vitro and biotin‐labelled *RNF115* 5′‐UTR transcription in vitro from Sangon. Briefly, 50 pmol of RNA was mixed with streptavidin magnetic beads (65602, Dynabeads MyOne streptavidin T1, ThermoFisher) in RNA capture buffer (1 mM EDTA, 1 M NaCl, 20 mM Tris pH 7.5) and incubated for 1 h. The cells were lysed using IP lysis buffer (150 mM KCl, Protease Inhibitor, RNase Inhibitor, 2 mM EDTA, .5 mM DTT, 10 mM HEPES pH 7.6, .5% NP‐40), centrifuged at 15 000 × *g*, and then added to the magnetic beads–RNA mixture and incubated in Protein–RNA Binding Buffer (2 mM MgCl_2_, .02 M Tris pH 7.5, .1% Tween 20, .05 M NaCl) for 1 h. Then, the magnetic beads were rinsed three times using Wash Buffer (10 mM NaCl, 20 mM Tris pH 7.5, .1% Tween 20). Loading buffer (Beyotime) was then added, and the mixture was heated at 100°C for 10 min before SDS‐PAGE analysis.

### 4.24 Xenograft assays

The null/null athymic BALB/c female mice (5–6 weeks old) were obtained from Beijing Vital River Laboratory Animal Technology Co., Ltd. and treated in compliance with the Animal Research Reporting of In Vivo Experiments (ARRIVE) guidelines. For xenograft implantation, SMMC‐7721(5 × 10^6^ cells) or Huh7 (8 × 10^6^ cells) sh‐NC, sh‐YBX1, sh‐RNF115 cells, Flag‐NC, Flag‐YBX1 cells were resuspended in PBS and injected subcutaneously into the right flank of each mouse. For the construction of subcutaneous xenografts of Huh7 cells overexpressing RNF115, 8 × 10^6^ cells were suspended in Matrigel (PBS: Matrigel = 1:1; Corning, 354262) and inoculated subcutaneously into BALB/c nude mice. After tumour volume to 100 mm^3^, mice were administrated with 5 mg/kg FIN56 with 98% corn oil and 2% DMSO via intraperitoneal injections (i.p.) once daily for consecutive 5 days, and then instead of every 2 days to give FIN56 (5 mg/kg) treatment. For Fer‐1 (5 mg/kg), every 2 days to give the abdominal injection. Tumour size was recorded every 2 days using callipers to measure the length (*L*) and width (*W*), and calculated using the following formula: *V* = (*L* × *W* ^2^)/2. At the endpoint of the experiment, the mice will be euthanised, and tumour tissues will be harvested for statistical analysis and subsequent experiments. The animal experiments were reviewed and approved by the Ethics Committee of the First Affiliated Hospital of Zhengzhou University (Zhengzhou, China).

### 4.25 DEN‐initiated HCC mouse model

The C57BL/6J mice (*n* = 5) were obtained from Beijing Vital River Laboratory Animal Technology Co., Ltd. and treated according to the ARRIVE guidelines. DEN (GLPBIO) 25 mg/kg was injected intraperitoneally when the mice were 2 weeks old. After at least 34 weeks, the mice were euthanised and the livers were harvested for subsequent analyses.

### 4.26 LC–MS/MS

The LC–MS/MS analyses were obtained with our previous workflow.^57^ The list of differential proteins after YBX1 knockdown is provided in Tables S3–S8.

### 4.27 RNA‐seq analysis

FastQ files were evaluated read quality by analysis with FastQC.^58^ Adapters were removed by Cutadapt and trimmed for read quality using Trimmomatic (phred ≥ 30).^59^, ^60^ The cleaning reads were mapped using Hisat2.^61^ The reads count generated from HTSeq were used as input data to downstream analysis with R package DESeq2.^62^, ^63^

### 4.28 Bis‐seq analysis

The m5C site analyses were obtained with previous workflow. Bis‐seq data from liver cancer were obtained from HRA001101.

### 4.29 RIP‐seq analysis

The raw data were filter by cutadapt and Trimmomatic.^59^, ^60^ The filter reads were aligned to Ensembl hg38 using Hisat2. Calling peaks were performed using Macs2.^64^ The peaks with adjusted *p*‐value ≤.05 and fold change ≥ 2 were recognised as YBX1 bound regions. Samtools was used to process any data files for visualisation.^65^

### 4.30 Ribo‐seq analysis

The raw data were checked using FastQC. The low‐quality reads were filtered by cutadapt and Trimmomatic. The filtered reads mapping to the rRNA transcriptome were removed. Using Bowtie and unmapped reads were retained for further analysis. After removal reads, the resulting reads were mapped to human genome (GRCh38) utilising STAR. The RPKM was calculated by Cufflinks. The translation efficiency was calculated by (FPKM of Ribo‐seq)/(FPKM of RNA‐seq).

### 4.31 Statistical analysis

The data are presented as the mean ± standard deviation (SD), unless indicated otherwise. Group comparisons were performed using a two‐tailed Student's *t*‐test. OS were analysed using the GEPIA, and *p* values were calculated using the two‐sided log‐rank test. A *p*‐value of *p < .05 was considered statistically significant.

## AUTHOR CONTRIBUTIONS

**Ouwen Li**: designed the study and wrote the manuscript. **Ke An** and **Jingyao Wei**: performed the bioinformatics analysis. **Han Wang**: constructed RNA‐seq, RIP‐seq and Ribo‐seq libraries. **Ouwen Li**, **Xianbin Li, Nuo Qin** and **Jiasheng Dong**: performed most of the experiments under the supervision of **Xin Tian**. **Yueqin Wang** and **Lan Huang**: provided scientific input and helped to edit the manuscript. **Yue Du** and **Yanjia Guo**: helped to edit the manuscript. **Ranran Sun** and **Xiangyi Sun**: constructed DEN‐initiated HCC mouse model. **Yong Shi**: provided assistance in some experiments. **Yun‐Gui Yang** and **Ying Yang**: provided the conception of the study. **Xin Tian** and **Quancheng Kan**: conceived the study.

## CONFLICT OF INTEREST STATEMENT

The authors declare no conflicts of interest.

## ETHICS STATEMENT

The current study was approved by the Ethics Committee of Scientific Research and Clinical Trial of the First Affiliated Hospital of Zhengzhou University (Approval 2022‐KY‐0005‐001), and complied with the Declaration of Helsinki. All subjects provided written informed consent according to the institutional guidelines.

## Supporting information



Supporting Information

Supporting Information

Supporting Information

Supporting Information

Supporting Information

Supporting Information

Supporting Information

Supporting Information

Supporting Information

## Data Availability

The sequencing data and other data that support the work are available from the corresponding author upon reasonable request.
