# Supplementary material for: Targeting YBX1‐m5C mediates RNF115 mRNA circularisation and translation to enhance vulnerability of ferroptosis in hepatocellular carcinoma
Source: Clin Transl Med. 2025 Mar 15;15(3):e70270. doi: 10.1002/ctm2.70270 (PMC11910144; doi:10.1002/ctm2.70270)
Supplement: Supplementary file 1 — Supporting Information [file CTM2-15-e70270-s005.docx]

**Targeting YBX1-m5C mediates *RNF115* mRNA circularization and translation to enhance vulnerability of ferroptosis in hepatocellular carcinoma**

Ouwen Li^1,2, #^, Ke An^1,2, #^, Han Wang^1,2, #^, Xianbin Li^1,2, #^, Yueqin Wang^1,2^, Lan Huang^3^, Yue Du^1,2^, Nuo Qin^1,2^, Jiasheng Dong^1,2^, Jingyao Wei^1,2^, Ranran Sun^4,5^, Yong Shi^1,2^, Yanjia Guo^1,2^, Xiangyi Sun^1,2^, Ying Yang^6^, Yun-Gui Yang^6, *^, Quancheng Kan^1,2, *^, Xin Tian^1,2, *^

1 Department of Pharmacy, the First Affiliated Hospital of Zhengzhou University, No.1 Jianshedong Rd, Zhengzhou, Henan, 450052, China.

2 Henan Key Laboratory of Precision Clinical Pharmacy, Zhengzhou University, Zhengzhou, 450052, China.

3 Translational Medicine Center, the First Affiliated Hospital of Zhengzhou University, Zhengzhou, Henan, 450052, China.

4 Department of Infectious Diseases, The First Affiliated Hospital of Zhengzhou University, Zhengzhou, Henan, 450052, China.

5 Gene Hospital of Henan Province, Precision Medicine Center, The First Affiliated Hospital of Zhengzhou University, Zhengzhou, Henan, 450052, China.

6 Key Laboratory of Genomic and Precision Medicine, Collaborative Innovation Center of Genetics and Development, Beijing Institute of Genomics, Chinese Academy of Sciences, China National Center for Bioinformation, Beijing 100101, China

# These authors contributed equally

*** Corresponding authors:** [tianx@zzu.edu.cn](mailto:tianx@zzu.edu.cn) (Xin Tian), [kanqc@zzu.edu.cn](mailto:kanqc@zzu.edu.cn) (Quancheng Kan), [ygyang@big.ac.cn](mailto:ygyang@big.ac.cn) (Yun-gui Yang)


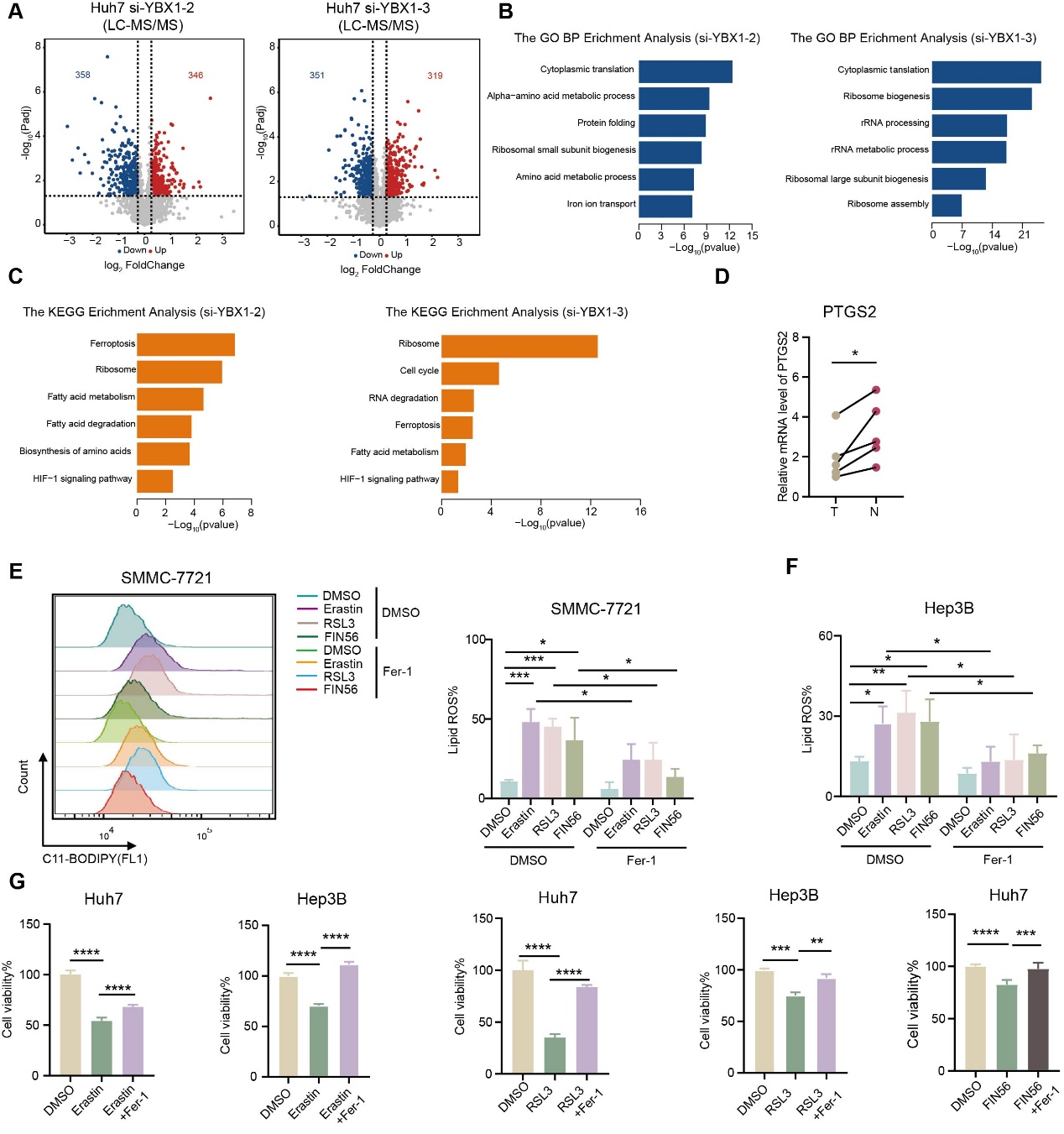


**Figure S1. Erastin, RSL3 and FIN56 induced ferroptosis in HCC cells.**

**(A-C)** In Huh7 cells, YBX1 was knocked down using si-YBX1-2/si-YBX1-3, followed by LC-MS/MS analysis. Volcano plot of differentially expressed proteins (A). GO and KEGG pathway enrichment analysis (B, C). **(D)** The expression level of *PTGS2* mRNA in the DEN-induced HCC model. **(E-F)** Lipid ROS levels were detected by flow cytometry in SMMC-7721 and Hep3B cells treated with Erastin (20 µM), RSL3 (10 µM), FIN56 (10 µM) or their combination with Fer-1 (1 µM) for 8 h. **(G)** Cell viability by CCK8 was detected after treatment with Erastin, RSL3, FIN56 or their combination with Fer-1 in Huh7 and Hep3B cells. Data are shown as mean ± SD, *p<0.05, **p<0.01, ***p<0.001, ****p<0.0001. Unpaired t test was used unless otherwise stated.


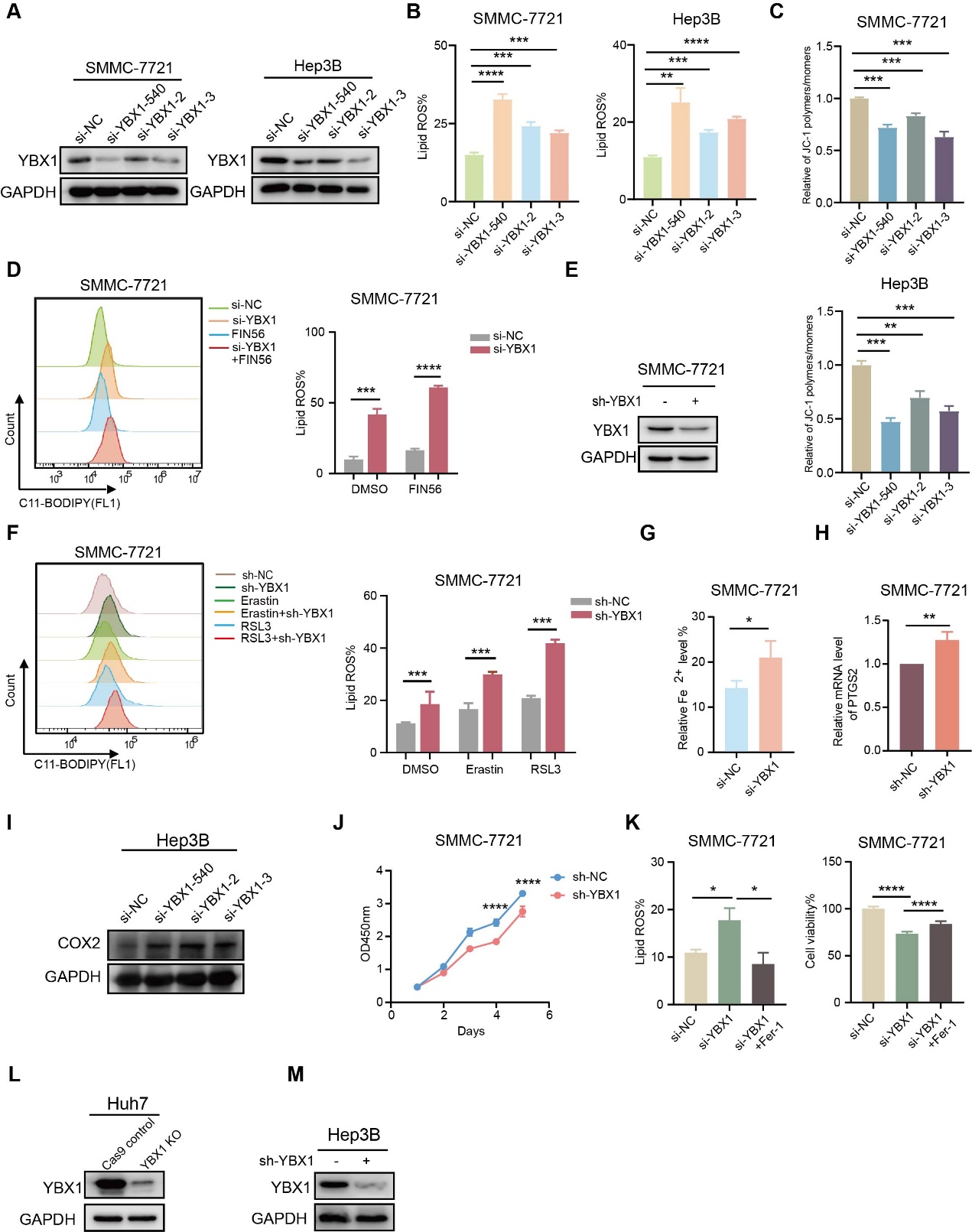


**Figure S2. Knockdown of YBX1 induces ferroptosis in HCC cells.**

**(A)** The efficiency of YBX1 knockdown in Hep3B and SMMC-7721 cells was verified by WB. **(B, C)** Lipid ROS and mitochondrial membrane potential were assessed by flow cytometry after knockdown of YBX1 in SMMC-7721 and Hep3B cells. **(D-F)** After knockdown YBX1 in SMMC-7721 cells and then lipid ROS was detected by flow cytometry after treatment with FIN56 (5 µM), Erastin (20 µM) or RSL3 (5 µM) for 8 h. **(G-J)** Fe^2+^ loading, *PTGS2* mRNA and COX2 protein levels and cell proliferation were detected after YBX1 knockdown. **(K)** After 48 hours of YBX1 knockdown in SMMC-7721 cells, combined treatment with Fer-1 (1 μM, 8 h) was used to assess lipid ROS levels via flow cytometry or were exposed to Fer-1 (200 nM) for 72 h and cell viability was detected by CCK8 assay. **(L)** YBX1 was knocked out by CRISPR Cas9 technology, and the expression of YBX1 was detected by WB. **(M)** Efficiency verification of YBX1 knockdown by shRNA in Hep3B. Data are shown as mean ± SD, *p<0.05, **p<0.01, ***p<0.001, ****p<0.0001. Unpaired t test was used unless otherwise stated.


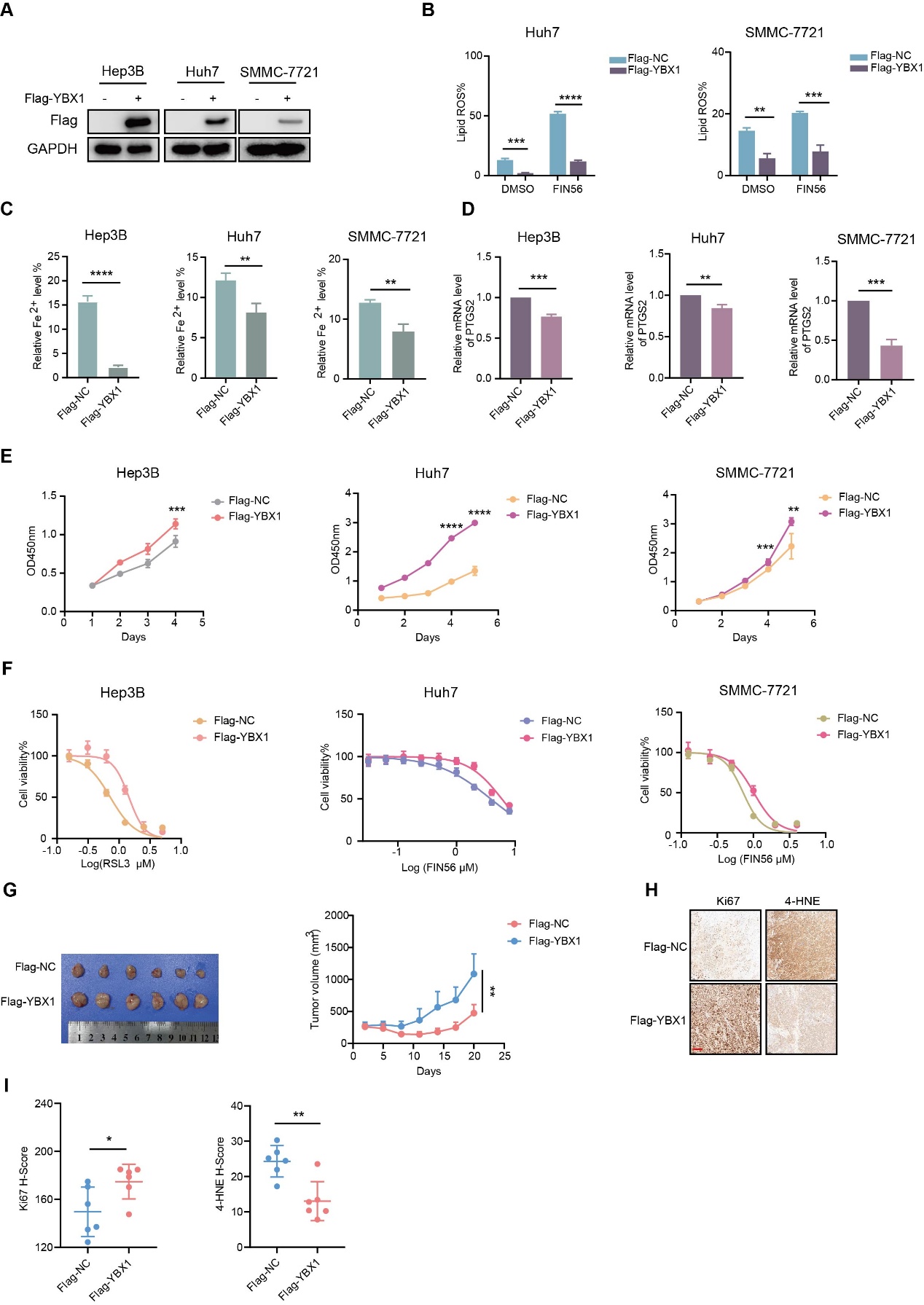


**Figure S3. Overexpression of YBX1 inhibited ferroptosis *in vitro*.**

**(A)** The efficiency of Flag-YBX1 overexpression in Hep3B, Huh7 and SMMC-7721 cells was verified. **(B)** Huh7 and SMMC-7721 cells stably transfected with Flag-YBX1 or Flag-NC were exposed to FIN56 for 8 h and lipid ROS was detected by flow cytometry. **(C-E)** Fe^2+^ loading, *PTGS2* mRNA levels and cell proliferation were detected in stable expression of Flag-YBX1 or Flag-NC cells. **(F)** CCK8 assay was used to detect the IC50 of RSL3 and FIN56 after overexpressing YBX1 in Hep3B (72 h), Huh7 (24 h) and SMMC-7721 (48 h) cells. **(G)** Tumor image and tumor volume of BALB/c nude mice subcutaneously implanted with Flag-NC-transfected or Flag-YBX1-transfected SMMC-7721 cells (n = 6 mice per group). **(H, I)** Representative images of Ki67 and 4-HNE IHC staining, along with the corresponding H-Score analysis, were obtained from tumor xenografts. Scale bar, 100 µm. Data are shown as mean ± SD, **p<0.01, ***p<0.001, ****p<0.0001. Unpaired t test was used unless otherwise stated.


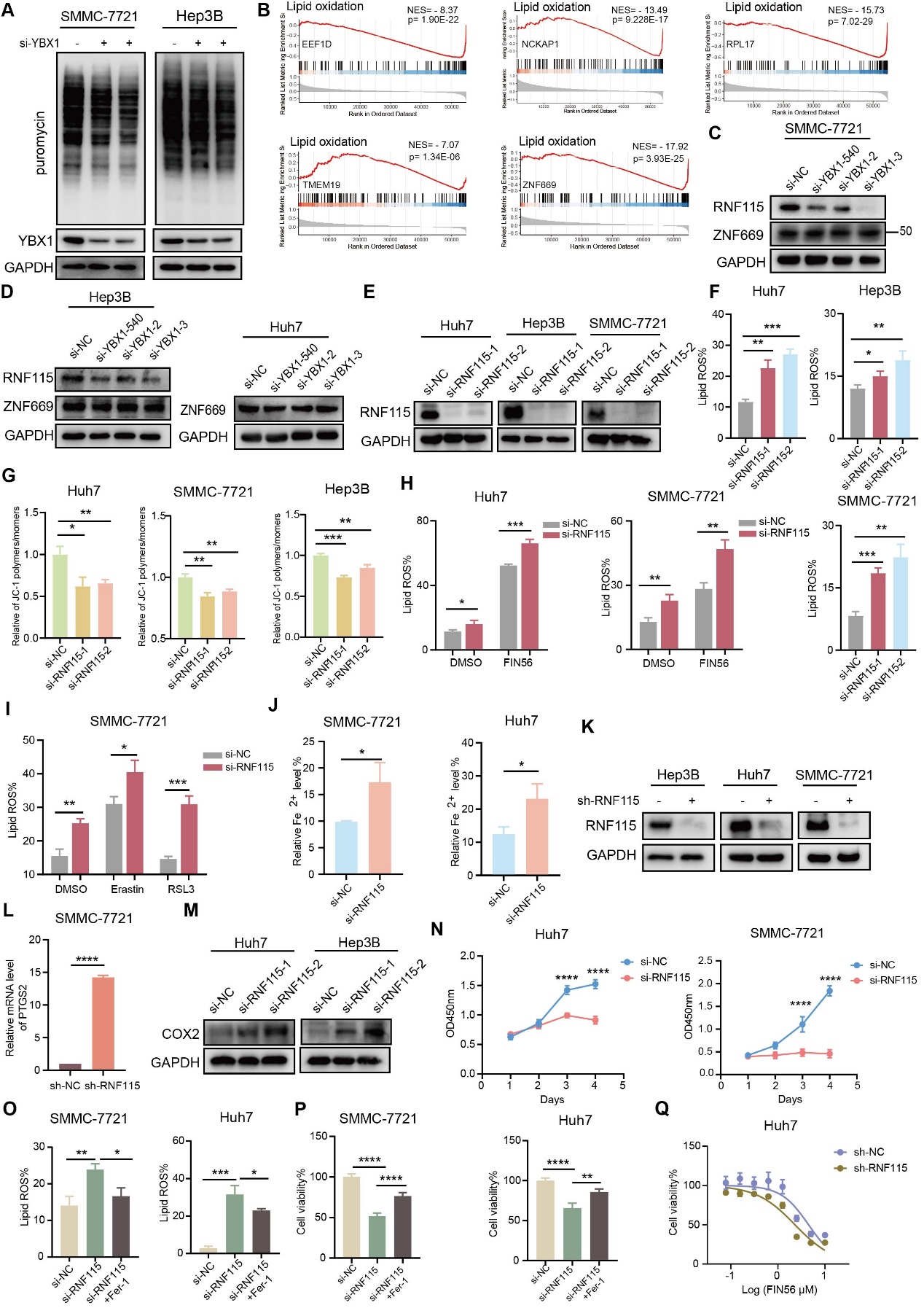


**Figure S4. Knockdown of RNF115 promotes ferroptosis in HCC.**

**(A**) SMMC-7721 and Hep3B cells transfected si-YBX1 for 48 h treated Puromycin (1 µM) for 1 h and protein synthesis was detected by WB. **(B)** Correlation analysis of EEF1D, NCKAP1, RPL17, TMEM19 and ZNF669 with lipid oxidation signaling by GSEA in TCGA database. **(C, D)** RNF115 and ZNF669 expression was detected by WB after 48 h of YBX1 knockdown by si-RNA. **(E)** Western blot analysis was used to evaluate the knockdown efficiency of RNF115. **(F, G)** Lipid ROS and mitochondrial membrane potential were assessed by flow cytometry after knockdown of RNF115. **(H, I)** Lipid ROS levels were detected by flow cytometry with or without treatment with FIN56 (5 µM, 8 h), Erastin (20 µM, 8 h) and RSL3 (5 µM, 8 h) after RNF115 knockdown by si-RNA for 48 h. **(J-N)** Fe^2+^ loading, RNF115 knockdown efficiency, *PTGS2* mRNA and COX2 protein levels and cell proliferation were detected after RNF115 knockdown. **(O, P)** Lipid ROS levels (O) and cell viability (P) were measured in SMMC-7721 and Huh7 cells with RNF115 knockdown with or without Fer-1 treatment. **(Q)** Huh7 cells stably transfected with sh-RNF115 were treated with FIN56 at gradient concentrations for 24 h and IC50 was measured by CCK8 assay. Data are shown as mean ± SD, *p<0.05, **p<0.01, ***p<0.001, ****p<0.0001. Unpaired t test was used unless otherwise stated.


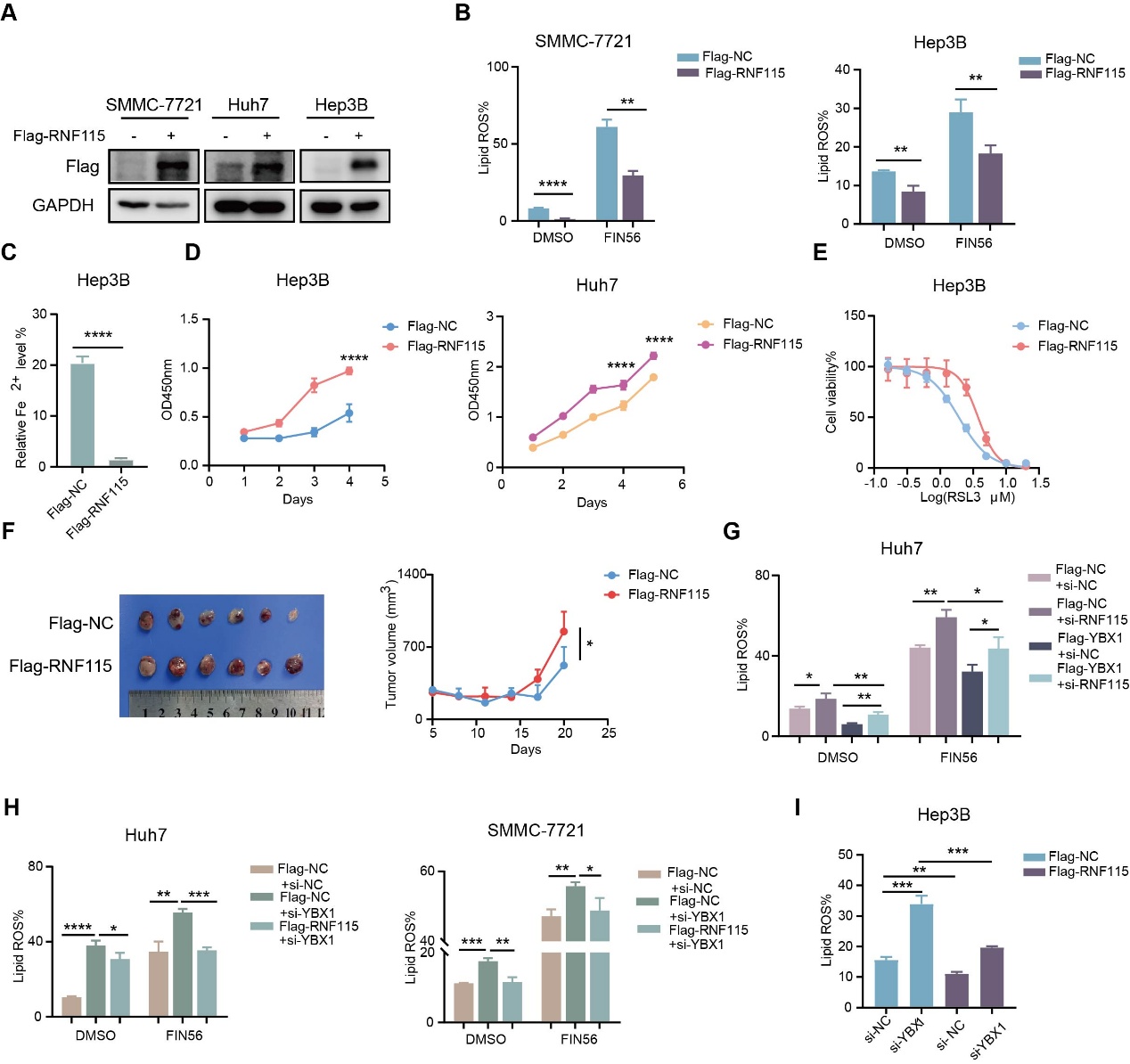


**Figure S5. YBX1 inhibits ferroptosis through RNF115.**

**(**A) The efficiency of stable overexpression of RNF115 was verified by WB. **(B)** SMMC-7721 and Hep3B cells stably transfected with Flag-RNF115 or Flag-NC were exposed to FIN56 for 8 h and lipid ROS was detected by flow cytometry. **(C-E)** Fe^2+^ loading, cell proliferation and RSL3 (72 h) IC50 were detected in stable expression of Flag-RNF115 or Flag-NC cells. **(F)** Tumor image and tumor volume of BALB/c nude mice subcutaneously implanted with Flag-NC-transfected or Flag-RNF115-transfected Huh7 cells (n = 6 mice per group). **(G)** Huh7 cells stably expressing Flag-NC or Flag-YBX1 were transfected with si-RNF115 for 48 h and then exposed to FIN56 (5 µM) for 8 h. Lipid ROS levels were detected by flow cytometry. **(H)** Huh7 and SMMC-7721 cells transfected with Flag-NC, si-YBX1 or si-YBX1 + Flag-RNF115 for 48 h exposed FIN56 (5 µM) for 12 h. **(I)** Hep3B cells transfected with Flag-NC, si-YBX1 or si-YBX1 + Flag-RNF115 for 48 h. Lipid ROS levels were detected by flow cytometry. Data are shown as mean ± SD, *p<0.05, **p<0.01, ***p<0.001, ****p<0.0001. Unpaired t test was used unless otherwise stated.


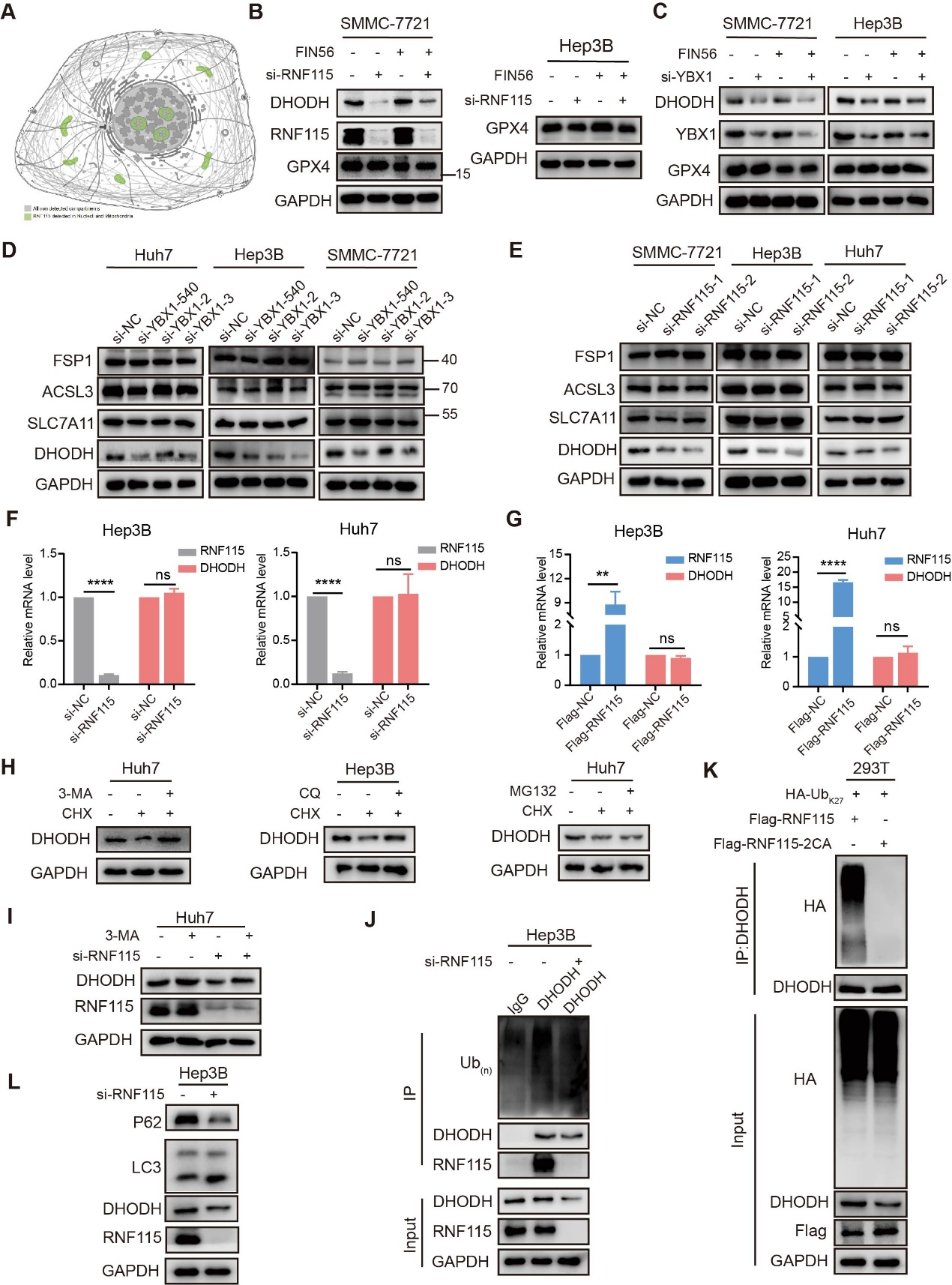


**Figure S6. RNF115 inhibits ferroptosis by mediating the K27 ubiquitination modification of DHODH.**

**(A)** Subcellular localization of RNF115 was analyzed using *The Human Protein Atlas* database. **(B, C)** After knockdown of RNF115 or YBX1 for 48 h by si-RNA, the expression of DHODH and GPX4 was detected by WB with or without FIN56 (2.5 µM, 24 h) treatment. **(D, E)** Ferroptosis-related proteins were detected by WB after knockdown of YBX1 or RNF115. **(F, G)** *DHODH* mRNA level was detected by qRT-PCR after knockdown or overexpression of RNF115. **(H)** After combined treatment with CHX (40 μM) and CQ (20 μM), 3-MA (5 mM) or MG132 (20 μM) for 24 h, the protein expression of DHODH was detected by WB. **(I)** After knockdown RNF115 in Huh7 cells and combined treatment with 3-MA (5 mM), the protein expression of DHODH was detected by WB. **(J)** After knockdown RNF115 in Hep3B cells, the ubiquitination level of DHODH was detected by Co-IP. **(K)** HEK293T cells were transfected with Flag-RNF115 or Flag-RNF115-2CA and HA-Ub_K27_ as indicated, the level of DHODH ubiquitination modification was detected by Co-IP. **(L)** WB was used to detect the expression levels of autophagy-related proteins after knockdown RNF115. Data are shown as mean ± SD, **p<0.01, ****p<0.0001, ns, not signification. Unpaired t test was used unless otherwise stated.


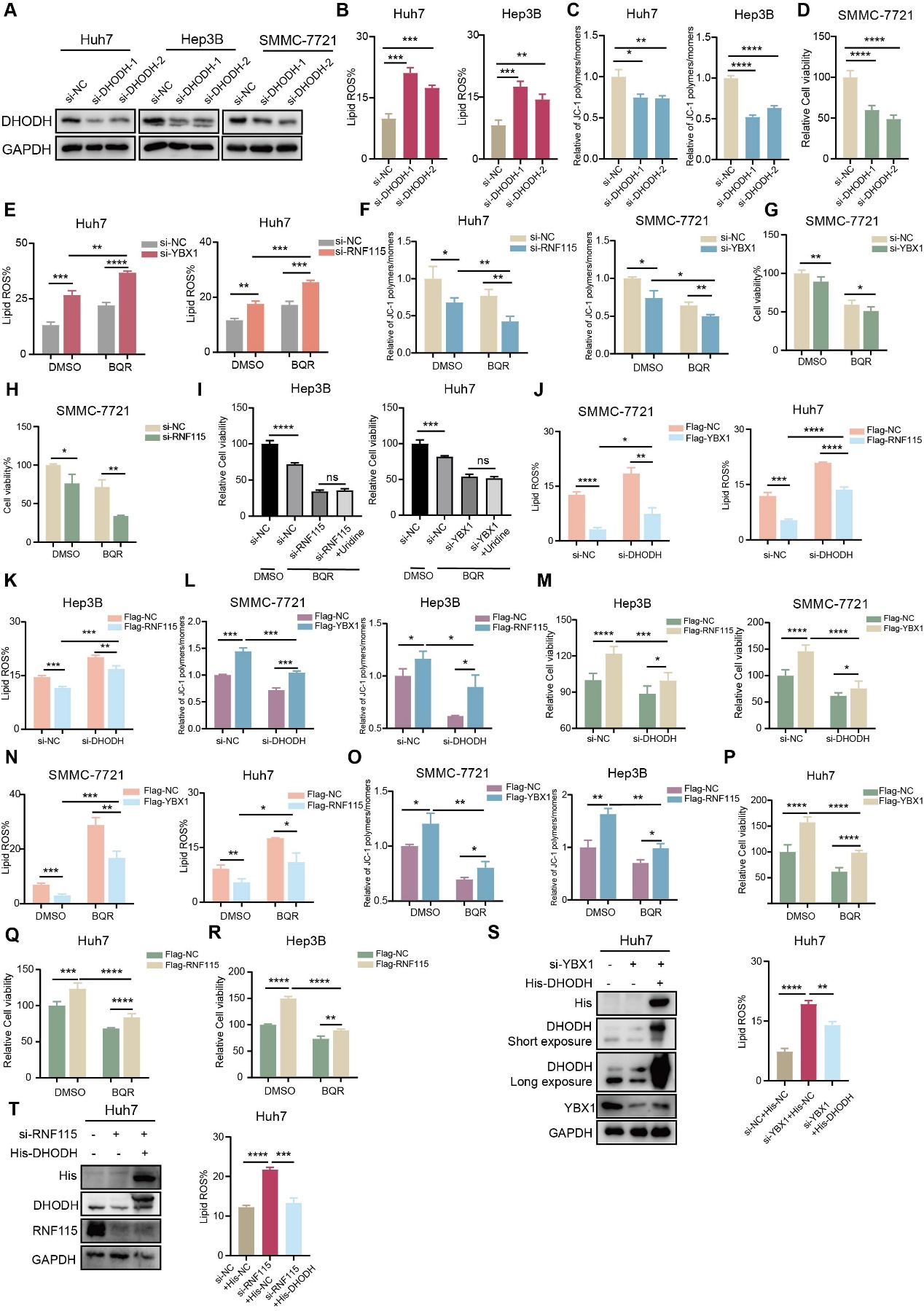


**Figure S7. YBX1/RNF115 resist ferroptosis in HCC via DHODH *in vitro*.**

**(A-D)** The knockdown efficiency of DHODH, lipid ROS, mitochondrial membrane potential, and cell viability were assessed after DHODH knockdown. **(E-H)** Lipid ROS (BQR, 20 µM for 24 h), mitochondrial membrane potential (BQR, 20 µM for 24 h), and cell viability (BQR, 5 µM for 48 h) were assessed after knockdown of YBX1/RNF115 combined with BQR treatment. **(I)** Cells transfected with si-RNF115 or si-YBX1 treated with BQR (5 µM) and Uridine (50 μM) for 48 h, and cell viability was detected by CCK8. **(J-M)** Lipid ROS, mitochondrial membrane potential, and cell viability were assessed after DHODH knockdown in cells stably expressing Flag-YBX1/Flag-RNF115. **(N-R)** Lipid ROS (BQR, 20 µM for 24 h), mitochondrial membrane potential (BQR, 20 µM for 24 h), and cell viability (BQR, 5 µM for 48 h) were assessed after BQR treatment in cells stably expressing Flag-YBX1/Flag-RNF115. **(S, T)** In Huh7 cells, YBX1/RNF115 were knocked down while His-DHODH was rescued. The rescue efficiency of DHODH was verified by Western blot, and lipid ROS levels were assessed by flow cytometry. Data are shown as mean ± SD, *p<0.05, **p<0.01, ***p<0.001, ****p<0.0001. Unpaired t test was used unless otherwise stated.


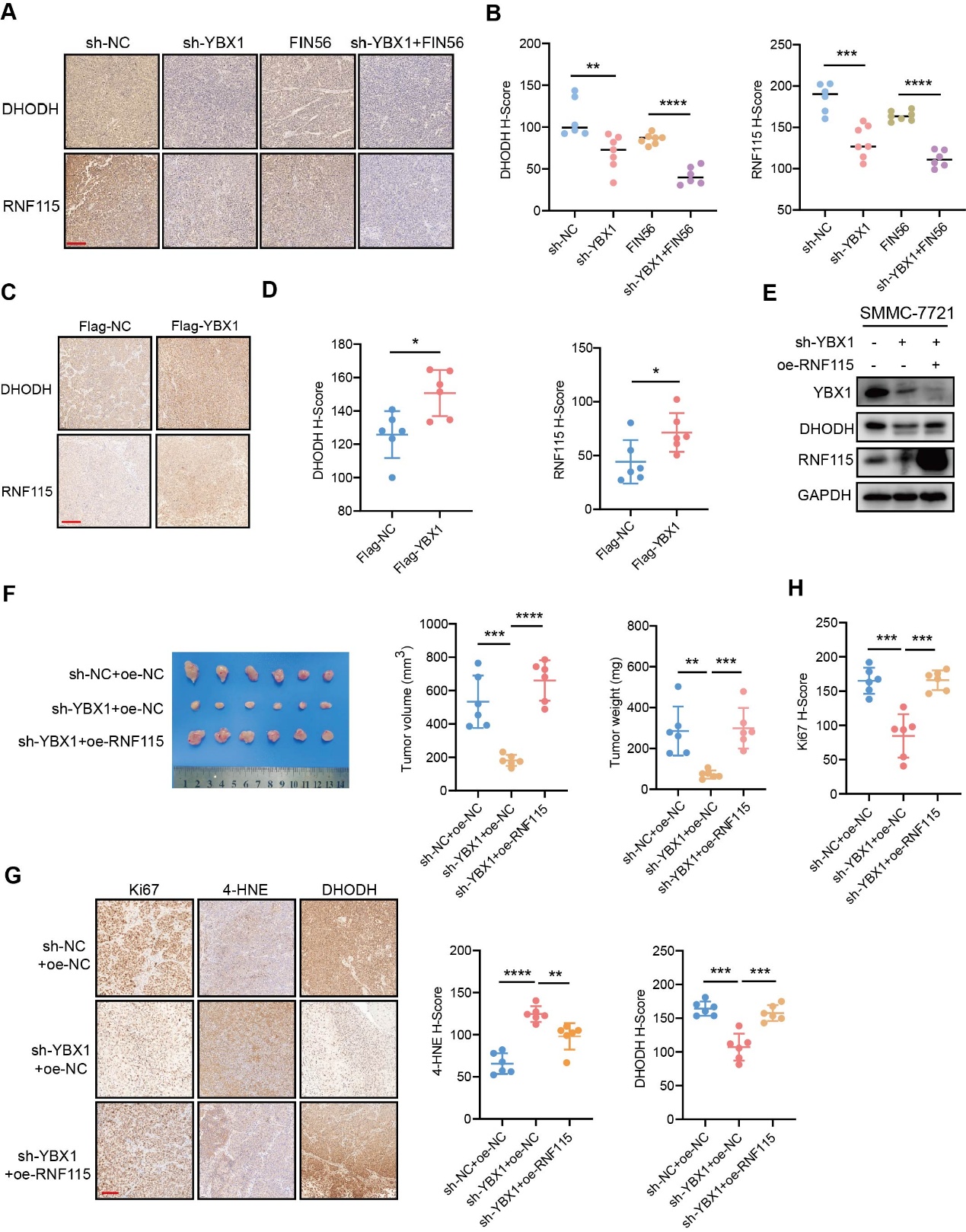


**Figure S8. YBX1/RNF115 resist ferroptosis in HCC via DHODH *in vivo*.**

**(A-D)** Representative images of RNF115 and DHODH IHC staining, along with the corresponding H-Score analysis, were derived from tumor xenografts. Scale bar, 100 µm. **(E)** The expression of DHODH was detected after knocking down YBX1 and restoring RNF115 expression. **(F)** *In vivo*, SMMC-7721 knocks down YBX1 and compensates for RNF115 to detect xenograft tumor volume and tumor weight. **(G, H)** Representative images of Ki67, 4-HNE, DHODH IHC staining, along with the corresponding H-Score analysis, obtained from (F) tumor xenografts. Scale bar, 100 µm. Data are shown as mean ± SD, **p<0.01, ***p<0.001, ****p<0.0001. Unpaired t test was used unless otherwise stated.


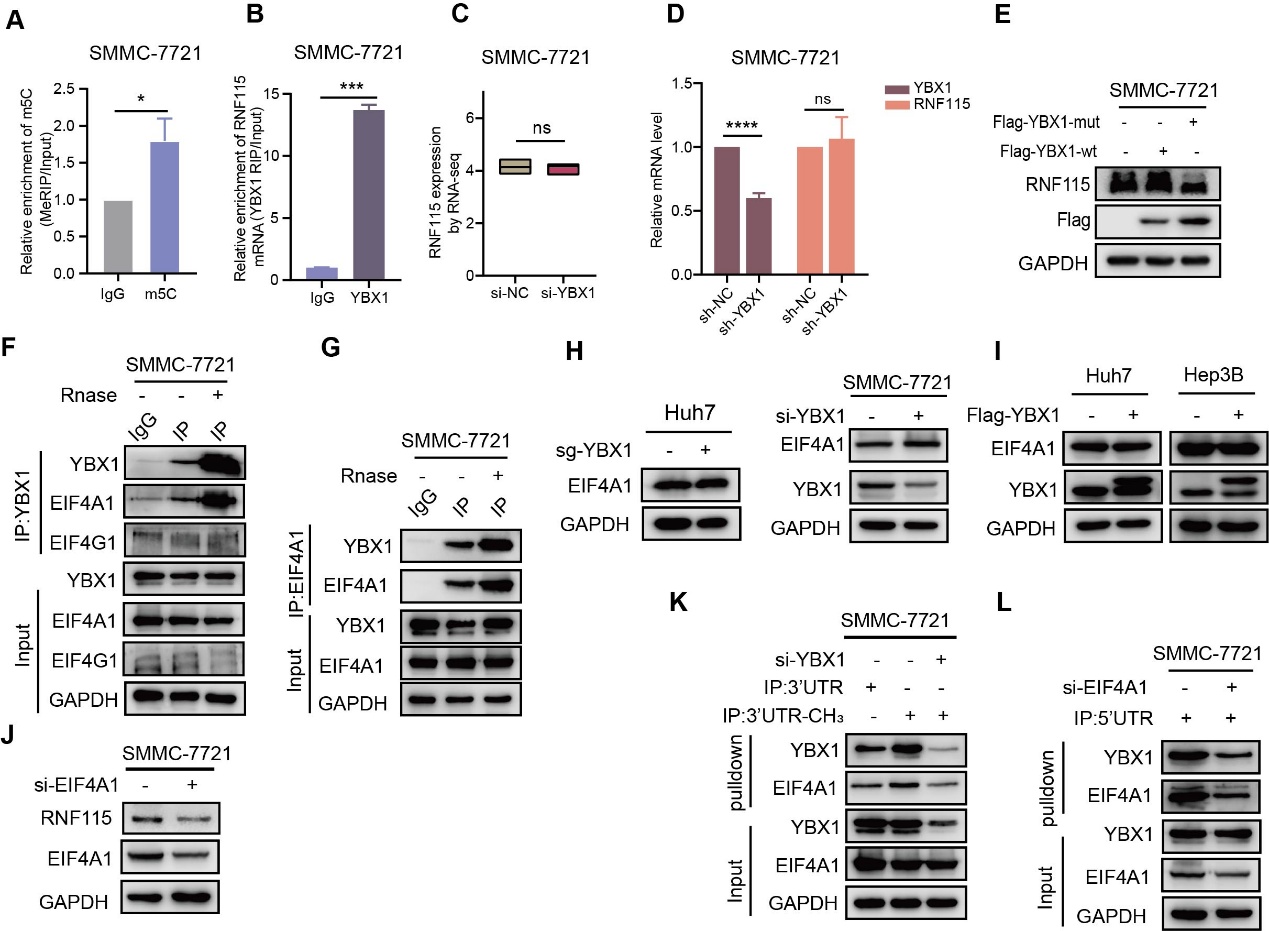


**Figure S9. YBX1 interacts with EIF4A1 to promote the circularization of *RNF115* mRNA.**

**(A**) MeRIP-qPCR was used to detect the m5C modification level of *RNF115* mRNA in SMMC-7721 cells, IgG was used as a control. **(B)** RIP-qPCR was used to detect the interaction between YBX1 and *RNF115* mRNA in SMMC-7721 cells, and IgG was used as a control. **(C, D)** After knockdown of YBX1, *RNF115* mRNA level was detected by RNA-seq or qRT-PCR. **(E)** Detect RNF115 protein levels in SMMC-7721 cells stably overexpressing YBX1-wt or YBX1-mut. **(F, G)** Co-IP analysis with or without Rnase (10 µg/ml, 37 ℃, 10 min) treatment in SMMC-7721 cells, IgG as a control. **(H)** After knockout of YBX1 in Huh7 cells or knockdown of YBX1 in SMMC-7721 cells, WB was used to detect the expression of EIF4A1. **(I)** The EIF4A1 expression was detected by WB in Huh7 and Hep3B cells stably expressing Flag-YBX1. **(J)** Knockdown of EIF4A1 in SMMC-7721 cells by si-RNA after 48 h, RNF115 expression was detected by WB. **(K, L)** RNA-pulldown to detect the interaction between YBX1, EIF4A1 and RNF115 3'-UTR or 5'-UTR after YBX1 or EIF4A1 knockdown in SMMC-7721 cells. Data are shown as mean ± SD, *p<0.05, ***p<0.001, ****p<0.0001, ns, not significant. Unpaired t test was used unless otherwise stated.


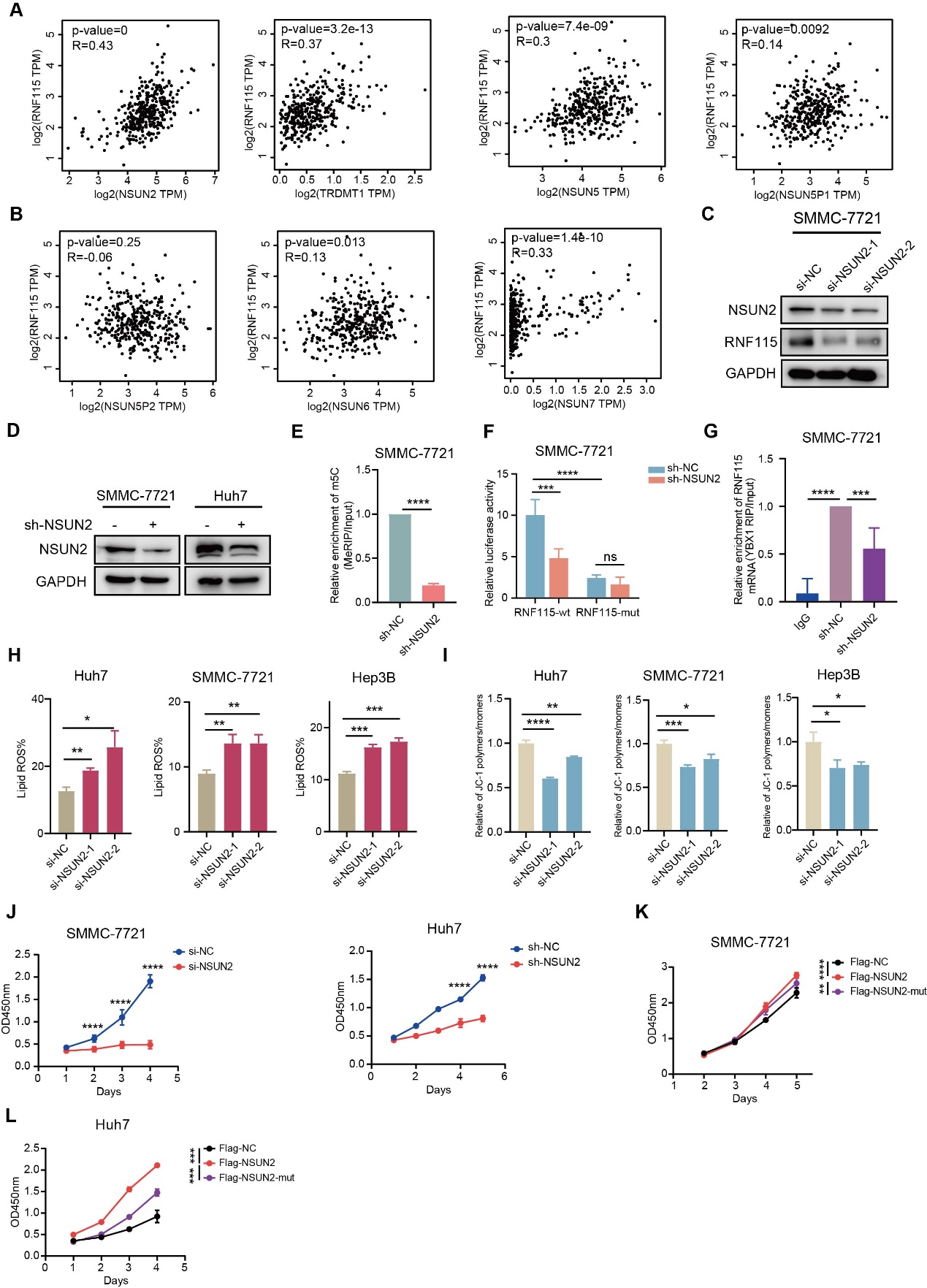


**Figure S10. NSUN2 inhibits ferroptosis by promoting RNF115 expression in an m5C-dependent manner.**

**(A, B)** Analyzed the correlation between RNF115 and m5C methyltransferases using the TCGA database. **(C)** The protein expression of RNF115 was detected after knockdown NSUN2 in SMMC-7721 cells. **(D)** Verification of efficient stable knockdown of NSUN2 in SMMC-7721 and Huh7 cells. **(E)** The m5C modification level of *RNF115* mRNA after NSUN2 knockdown was detected by MeRIP-qPCR. **(F)** Relative luciferase activity of the wild-type and mutant forms of RNF115 3'-UTR reporter vectors in SMMC-7721 cells stably transfected with sh-NC or sh-NSUN2, respectively. **(G)** After knockdown of YBX1, the binding level of YBX1 and *RNF115* mRNA was detected by RIP-qPCR. **(H, I)** Lipid ROS and mitochondrial membrane potential were assessed by flow cytometry after knockdown of NSUN2. **(J-L)** CCK8 assay was used to detect the proliferation ability of cells with knockdown of NSUN2 or overexpression of Flag-NSUN2 and Flag-NSUN2-mut. Data are shown as mean ± SD, *p<0.05, **p<0.01, ***p<0.001, ****p<0.0001, ns, not signification. Unpaired t test was used unless otherwise stated.
